# Guidelines for Essential Trauma Care: Second Edition (2026)

**DOI:** 10.1002/wjs.70321

**Published:** 2026-04-20

**Authors:** Charles Mock, Timothy C. Hardcastle, Christine Gaarder, Amit Gupta, Adam Gyedu, Manjul Joshipura, Elmin Steyn, the Essential Trauma Care revision author group, Valencia Adjei, Roger Alcock, Vanda Amado, Michael Boampong, Sanjeev Booi, Petra Brysiewicz, Corazon Cabanilla‐Manuntag, Cristiane de Alencar Domingues, Ghada Elhadari, Ranjith Nanda Ellawala, Sarnai Erdene, Ama Akoma Essuman, Jorge Esteban Foianini, Filippo Gatti, Armando Garcia Guererra, Greg Hynes, Adil Haider, Sandy Ingless, Balavenkata Jagannathan, Helen Jowett, Knut Magne Kolstadbråten, Sonja Krofak, Aireen Patricia Madrid, Tina Tomić Mahečić, Claude Martin, Daniel Ntogwrachu, Bisola Onajin‐Obembe, Nhobojit Roy, Andres Rubiano, Niisoja Mensah Torto, Ben Wachira, Meg Walmsley, Torben Wisborg, Elma Wong

**Affiliations:** ^1^ Department of Surgery University of Washington Seattle Washington USA; ^2^ Department of Surgical Sciences Nelson R Mandela School of Medicine Faculty of Health Sciences University of KwaZulu‐Natal Durban South Africa; ^3^ Inkosi Albert Luthuli Central Hospital Durban South Africa; ^4^ Department of Gastro‐intestinal Surgery and Trauma Oslo University Hospital Oslo Norway; ^5^ Division of Trauma Surgery and Critical Care JPN Apex Trauma Centre All India Institute of Medical Sciences New Delhi India; ^6^ Department of Surgery School of Medical Sciences Kwame Nkrumah University of Science and Technology Kumasi Ghana; ^7^ Academy of Traumatology (India) Ahmedabad India; ^8^ Division of Surgery Faculty of Medicine and Health Sciences Stellenbosch University Cape Town South Africa

**Keywords:** global, injury, low‐ and middle‐income country, resources, trauma

## Abstract

Injury is a major cause of death and disability globally, with the highest burden in low‐ and middle‐income countries (LMICs). Strengthening the organization and planning for trauma care (care of the injured) can improve care and lower mortality. In 2004, the International Association for Trauma Surgery and Intensive Care (IATSIC) and the World Health Organization (WHO) co‐published the *Guidelines for Essential Trauma Care* (*EsTC*). The goals of the *Guidelines for EsTC* were to promote affordable and achievable standards for trauma care resources that could realistically be achievable at health care facilities anywhere in the world, even in the lowest‐income settings. By so doing, IATSIC and WHO hoped to strengthen trauma care services globally, especially in LMICs. Since its publication in 2004, the *Guidelines for EsTC* have been extensively cited. More importantly, there have been documented, published examples of implementation of the *Guidelines* in 48 countries worldwide, spanning all economic levels from low‐income to high‐income countries. The current publication represents the first update and revision of the *Guidelines for EsTC*. As with the first edition, the current edition contains resource tables listing human resources (skills, training, staffing) and physical resources (equipment and supplies) that should be available at varying health care facilities in all countries, ranging from clinics to first‐level hospitals to second‐level hospitals to tertiary hospitals. These resource tables cover the breadth of trauma care, including initial management and resuscitation to definitive care of most major injuries. As with the original version, these resource tables are meant to be flexible to allow adjustments as needed to tailor them based on local health care system resources and capabilities. The *Guidelines* focus on fixed facilities, as other publications address prehospital care.

## Graphical abstract

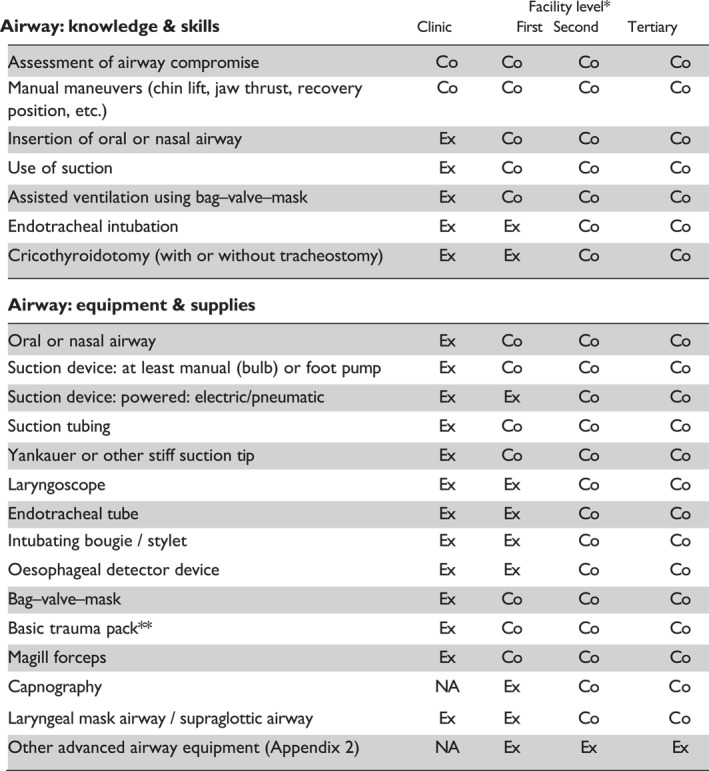

## 1 Executive Summary

Injury is a major cause of death and disability globally, with the highest burden in low‐ and middle‐income countries (LMICs). Strengthening the organization and planning for trauma care (care of the injured) can improve care and lower mortality. In 2004, the International Association for Trauma Surgery and Intensive Care (IATSIC) and the World Health Organization (WHO) co‐published the *Guidelines for Essential Trauma Care* (*EsTC*). The goals of the *Guidelines for EsTC* were to promote affordable and achievable standards for trauma care resources that could realistically be achievable at health care facilities anywhere in the world, even in the lowest‐income settings. By so doing, IATSIC and WHO hoped to strengthen trauma care services globally, especially in LMICs.

Since its publication in 2004, the *Guidelines for EsTC* have been extensively cited. More importantly, there have been documented, published examples of implementation of the *Guidelines* in 48 countries worldwide, spanning all economic levels from low‐income to high‐income countries.

The current publication represents the first update and revision of the *Guidelines for EsTC*. As with the first edition, the current edition contains resource tables listing human resources (skills, training, staffing) and physical resources (equipment and supplies) that should be available at varying health care facilities in all countries, ranging from clinics to first‐level hospitals to second‐level hospitals to tertiary hospitals. These resource tables cover the breadth of trauma care, including initial management and resuscitation to definitive care of most major injuries. As with the original version, these resource tables are meant to be flexible to allow adjustments as needed to tailor them based on local health care system resources and capabilities. The *Guidelines* focus on fixed facilities, as other publications address prehospital care.

This update was developed by IATSIC, with input and advice from WHO staff members and with use of several WHO publications on emergency care, surgical care, resources for humanitarian care, essential medicines, among others. It was also developed with input from the Trauma Care Resource Consensus Group, convened specifically for this revision and consisting of 36 experts from all continents and from a wide breadth of professional backgrounds. The revision reflects the solid trauma care principles found in other IATSIC resources, such as its National Trauma Management Course and its Definitive Surgical Trauma Care Course.

The target audience for these *Guidelines* includes: ministry of health planners, hospital administrators (including nursing service directors and department/medical service directors), and trauma care clinicians including surgeons of all sub‐specialties, emergency physicians, anesthetists, nurses, and other professionals who care for the injured. It is intended that planners and administrators will use the *Guidelines* in developing trauma services and allocating resources for these services. It is also intended that clinicians will use these *Guidelines* as a minimum standard to identify and demonstrate to planners and administrators key gaps that need to be addressed.

The *Guidelines* conclude with consideration of key administrative functions that should be active at health care facilities to assist with achieving the resource requirements and to assure the quality of trauma care. These include: quality improvement programs, trauma registries, use of continuing professional development courses, triage systems, trauma teams, tools (e.g., checklists), and trauma care protocols.

Finally, it is interesting to note how the changes in resource designations since the 2004 version reflect improvements in trauma care capabilities globally. The expert consensus process used to develop the *Guidelines* in both 2004 and currently provides a snapshot of what global experts consider feasible to promote, across diverse settings, even in the most resource‐constrained circumstances. Due in part to increased attention to trauma care, improved economic standards in many countries, and lower costs for some items of technology, several critical items that were not recommended or were recommended only at tertiary levels previously, are now recommended to be much more widely applicable at many other levels of the health care system. These include (among others) pulse oximetry, mechanical ventilation, ultrasonography for diagnosis of intra‐abdominal bleeding, and laboratory facilities for measurement of electrolytes and arterial blood gases. The developers of the current version of the *Guidelines for EsTC* hope and intend that this version will be used as extensively as the 2004 version and will achieve similar improvements in trauma care capabilities globally.

### 1.1 Glossary of Abbreviations

**Table:** 

BP	Blood pressure
Co	Core (in Tables 1, 2, 3A, 4, 5, 6, 7, 8, 9, 10, 11, 12, 13, 14, 15)
CPD	Continuing professional development
CT	Computerized tomography
DALY	Disability adjusted life years
DPL	Diagnostic peritoneal lavage
EsTC	Essential Trauma Care
Ex	Extended (in Tables 1, 2, 3A, 4, 5, 6, 7, 8, 9, 10, 11, 12, 13, 14, 15)
FAST	Focused assessment with sonography for trauma
IATSIC	International Association for Trauma Surgery and Intensive Care
ICP	Intracranial pressure
ICU	Intensive care unit
IV	Intravenous
LMIC	Low‐ and middle‐income country
MLEM	Model List of Essential Medicines
MRI	Magnetic resonance imaging
NA	Not applicable
QI	Quality improvement
The “*Guidelines for EsTC*”	The *Guidelines for Essential Trauma Care*
The “*Guidelines*”	The *Guidelines for Essential Trauma Care*
WHO	World Health Organization

## 2 Orientation to the *Guidelines for Essential Trauma Care*

### 2.1 Need for the *Guidelines*

Injury is one of the leading causes of death and disability globally. Each year, it accounts for 4.4 million deaths (8% of all deaths globally) [1] and 268 million disability adjusted life years (DALYs) lost (10% of all DALYs globally) [2, 3]. The vast majority (over 80%) of all injury related deaths occur in low‐ and middle‐income countries (LMICs). Rates of injury‐related death are highest in low‐income countries (78/100,000/year) and in lower‐middle‐income countries (58) than in upper‐middle‐income (51) and high‐income countries (53) [1, 2, 3, 4].

Part of the reason for the higher injury death in LMICs is more unsafe conditions, such as higher rates of road traffic crashes. Part of the reason is because of higher case‐fatality rates in LMICs once someone is injured. Several studies have documented such increased rates. For example, a study from India showed mortality for hospitalized patients to be eight times higher than for hospitalized patients in the USA (23.2% vs. 2.8%), despite the fact that the groups had similar degrees of injury (both with median Injury Severity Score—ISS—of 9) [5]. Similarly, looking at both prehospital and hospitalized patients with severe injuries (ISS of 9 or more), mortality rates rose from 35% in a high‐income country to 55% in a middle‐income country to 63% in a low‐income country [6]. Eliminating these inequalities in case‐fatality rates could save one million lives per year [7].

Contributing to the above discrepancies in case‐fatality rates for injured patients are limitations in resources for trauma care (care of the injured) in LMICs. These include limitations of human resources (staffing, training) and physical resources (equipment, supplies) for trauma care. Studies from numerous LMICs have documented shortcomings in the availability of many trauma care services and the resources needed to provide them. These include high‐cost items (e.g., computerized tomography—CT scans). But, there are also shortages in many lower‐cost items, such as airway equipment and IV fluids, which are often absent not because of their cost but because of shortcomings in organization and planning [8, 9, 10, 11, 12, 13]. It is important to emphasize that much of trauma care is very affordable and cost‐effective. A range of trauma care services in several LMIC hospitals has been estimated to cost $87–$302 per DALY averted. This compares very favorably to several widely used public health measures such as oral rehydration solution (US$1000 per DALY averted) and antiretroviral therapy for HIV/AIDS (US$900 per DALY averted) [7, 14, 15].

In addition to human and physical resources, there is a dearth of administrative mechanisms to assure the quality of trauma care at most LMIC hospitals, including quality improvement programs, trauma registries, trauma teams with pre‐assigned roles, and transfer protocols (among others). As with the physical resources noted above, most of these mechanisms are very low‐cost.

Addressing such deficiencies has been the goal of several World Health Assembly resolutions on trauma care, and on surgical care and emergency care more generally. These resolutions have also encouraged the development of minimum standards for trauma and other emergency care services and the resources needed to provide them [16, 17, 18, 19].

### 2.2 History and Use of the *Guidelines for Essential Trauma Care*

In order to promote improved organization and planning for trauma care services globally, the International Association for Trauma Surgery and Intensive Care (IATSIC) and the World Health Organization (WHO) co‐published the *Guidelines for Essential Trauma Care* (*EsTC*) in 2004 [20]. The goal of the *Guidelines for EsTC* was to better define what essential trauma treatment (care) services should realistically be made available to almost every injured person worldwide. It then defined the human resources (training and staffing) and physical resources (supplies and equipment) necessary to provide these essential trauma care services, even in the lowest income settings. It created a flexible matrix of over 200 elements of human and physical resources that was intended to serve as a template to be used as a guide for those planning trauma care services for their countries or areas, including both clinicians and administrators. The recommendations of the *Guidelines* were intended to be practical and to be realistically achievable within the confines of tight health care budgets in most LMICs. The *Guidelines* addressed care for a range of injuries, including those arising from blunt trauma (e.g., road traffic crashes, falls), penetrating trauma (e.g., gunshot wounds, stab wounds), and burns. Many of the resources that it addresses are also important for the treatment of a wide range of other emergency conditions, such as those for treatment of obstructed airways, hypoxia, and shock.

The 2004 *Guidelines for EsTC* have been widely implemented around the world. In 2016, a systematic review was published on this implementation [21] (covering 2004–May 2015). In preparation for this current revision of the *Guidelines*, the systematic review was updated (covering June 2015 to April 2025), with the results contained herein. Altogether (2004–2025), 117 separate instances of implementation of the *Guidelines for EsTC* have been published, covering 48 countries across the economic spectrum (from low‐income to high‐income), in addition to regional and global implementation [21] (Appendix 1). The majority of these have been needs assessments (*n* = 61) in which the *Guidelines for EsTC* were used as a template against which to compare the human and physical resources available at clinics and hospitals in given area or country.

In two countries (Ghana and Vietnam), these needs assessments served as stimuli to promote improvements, which were documented by subsequent studies [22, 23, 24]. In Vietnam, the Hanoi Health Department runs a network of clinics (commune health stations), first‐level, and second‐level hospitals. Clinicians working in the health department used the *Guidelines for EsTC* to evaluate the availability of 12 items of human and physical resources at the network of facilities. This identified deficiencies in several low‐cost resources. Advocacy by the clinicians helped to bring these deficiencies to the attention of health department leaders. As most of the items were low‐cost, addressing them was primarily a matter of attention to detail in planning, rather than cost. Repeat surveys over the next several years documented improvements, with nine of 12 items (e.g., basic airway equipment) improving in availability at the clinics and almost all of the 12 items improving at both hospital levels [22, 23]. A similar study in Ghana used the *Guidelines* to document changes in trauma care capacity over a 10‐year period, during which there had been several initiatives that encompassed trauma care nationwide. Advocacy related to the findings of the essential trauma care survey was one of these. The essential trauma care surveys also provided an internationally‐recognized metric to use to monitor changes. Changes over time included improvements in most (but not all) resources evaluated. For example, oxygen availability increased from under 50% to nearly 100% at second‐level hospitals [24].

Other types of implementation events have included stakeholder endorsements (*n* = 25), in which groups (especially country professional societies) have formally endorsed the *Guidelines* and called on their governments to utilize them (e.g., the Academy of Traumatology (India) and the Ecuadorian Trauma Society). Smaller numbers of instances of implementation have included: use in educational interventions (*n* = 14) and policy development (*n* = 14). Examples of educational interventions include that the *Guidelines* were incorporated into educational modules for humanitarian aid workers in Burkina Faso and Sierra Leone and for emergency nurses in Sri Lanka. An example of policy implementation includes that the *Guidelines* were cited and utilized in the development of regulations on emergency care in Mexico. It should be noted that the above implementation events are only those that were found by a literature search. There are likely many more implementation events that were not published. Finally, the low number of episodes of incorporation into policy indicates a barrier to overcome in future efforts to implement the *Guidelines*.

### 2.3 Update Process

The *Guidelines for EsTC* have now been updated in 2026. A working group of IATSIC members, with input from WHO staff, made preliminary updates to the 2004 resource grids, including changing designations of resources as to whether they were core, extended, or not applicable at different levels of the health care system (more in Section 4) and adding new resources that were not covered in the 2004 edition. These were then reviewed and commented on by the Trauma Care Resource Consensus Group which consisted of 35 members from all continents and from countries at all economic levels. This group came from a wide variety of professional backgrounds involved in care of the injured, including: anesthesia, critical care, emergency medicine, family medicine/general doctors (especially those working at first level hospitals), neurosurgery, nursing (including critical care nursing and trauma nursing), pediatric surgery, rehabilitation, and trauma surgery (both general surgery and orthopedics). The consensus group included members of IATSIC and members of several of the professional societies that are members of WHO's Global Alliance for Care of the Injured (GACI): including African Federation of Emergency Medicine, AO Alliance, G4 Alliance, International Committee of the Red Cross, International Federation of Emergency Medicine, International Federation of Red Cross and Red Crescent Societies, Médecins Sans Frontières, Panamerican Trauma Society, Society of Trauma Nurses, and the World Federation of Critical Care Nurses.

The preliminary changes made by the IATSIC working group were amended as needed based on input from the consensus group. The resource assignments took into account and were harmonized as needed with several WHO resources, including the Model List of Essential Medicines [25], high‐priority health services for humanitarian response (H3 package) [26], essential resources for emergency and critical care (ERECC), Universal Health Coverage (UHC) Service Package Delivery and Implementation (SPDI) Platform [27], and the Basic Emergency Care course [28]. The updates also took into account the solid trauma care principles found in other IATSIC resources, such as its National Trauma Management Course and its Definitive Surgical Trauma Care Course [29]. After completion of the first draft of the revision, independent review was obtained from nine people experienced in various aspects of trauma care in LMICs and who had not been involved in the revision process. Their input was used to further refine the *Guidelines*.

### 2.4 Orientation to the Rest of This Publication

As with the original 2004 version of the *Guidelines*, the current version starts with a list of medical goals that should be feasible for most injured persons everywhere. These can be viewed as the “needs of the injured patient” (chapter 3). In order to assure the achievement of such goals, the inputs of human and physical resources must be utilized in an optimal process. To this end, the authors have developed a template for the resources that are needed. These are described in chapters 4 and 5. The authors envision that this template will be used as a guide for those planning trauma treatment services. These *Guidelines* will hopefully be of relevance to planners in ministries of health, to hospital administrators, to directors of trauma services, to nursing service directors and to clinicians, both individually and collectively, through organizations such as societies of surgery, anesthesia, emergency medicine, traumatology and other disciplines that deal with the injured patient. These groups constitute the target audience for the *Guidelines.* The *Guidelines* focus on resource planning for individual facilities. This is part of broader trauma system planning encompassing prehospital care, interfacility transfer, and referral networks [30, 31, 32].

## 3 Essential Trauma Services: Rights of the Injured

This section contains a list of those services which the authors feel are essential to prevent death and disability in injured patients. They might be considered as the “needs of the injured patient.” IATSIC endorses these as the “rights of the injured” and as the responsibility of governments to provide to their people.

These can be categorized into three broad sets of needs:

1. Life‐threatening injuries are appropriately treated, promptly and in accordance with appropriate priorities, so as to maximize the likelihood of survival.
2. Potentially disabling injuries are treated appropriately, so as to minimize functional impairment and to maximize the return to independence and to participation in community life.
3. Pain and psychological suffering are minimized.

Within these three broad categories, there are several specific medical goals that are eminently achievable within the resources available in most countries.

- Obstructed airways are opened and maintained before hypoxia leads to death or permanent disability.
- Impaired breathing is supported until the injured person is able to breathe adequately without assistance.
- Pneumothorax and hemothorax are promptly recognized and relieved.
- Bleeding (external or internal) is promptly stopped.
- Shock is recognized and treated with intravenous (IV) fluid replacement before irreversible consequences occur.
- The consequences of traumatic brain injury are lessened by timely decompression of space occupying lesions and by prevention of secondary brain injury.
- Intestinal and other abdominal injuries are promptly recognized and repaired.
- Potentially disabling extremity injuries are corrected.
- Potentially unstable spinal cord injuries are recognized and managed appropriately, including early immobilization.
- The consequences to the individual of injuries that result in physical impairment are minimized by appropriate rehabilitative services.
- Medications for the above services and for the minimization of pain are readily available when needed.

The precise procedures that can optimally be applied to achieve these goals, as well as the human and physical resources needed to optimally carry out these procedures, will vary across the spectrum of economic resources of the countries of the world and the geographic location of the facilities concerned. However, these goals should be achievable for most injured patients in most locations. It should also be noted that these goals apply to the entire system and not necessarily all individual facilities. Some of the goals are applicable to all levels of the health care system, especially as regards airway, breathing, and circulation. Some of the above goals would require timely referral from smaller facilities with definitive care mostly be provided at higher level facilities (e.g., decompression of space occupying lesions after head injuries). These details are spelled out in later sections of the *Guidelines for EsTC*.

The provision of these services should not be dependent on ability to pay. Hence, cost recovery schemes, necessary though they may ultimately be, should not preclude the provision of initial emergency care nor of critical elements of definitive care.

## 4 Inputs Needed to Achieve Essential Trauma Services

### 4.1 Overview

The goals outlined in the previous chapter depend on the provision of specific items of physical examination, diagnostic tests, medications and therapeutic procedures. Likewise, the ability of the health system to provide these items depends on the inputs of human resources (training and staffing) and physical resources (equipment and supplies). The following sections of these *Guidelines for EsTC* outline those resources which the authors feel are essential to the provision of essential trauma services. These resources are outlined in the form of the resource matrix for essential trauma care (Tables 1, 2, 3A, 3B, 4, 5, 6, 7, 8, 9, 10, 11, 12, 13, 14, 15).

### 4.2 Resource Matrix: Introduction

The resource matrix for trauma care contains brief descriptions of the resources that need to be available for the provision of specific categories of care at different levels of the health care system. A specific matrix is derived for each of 15 categories of care, such as airway, shock, head injuries, extremity injuries and rehabilitation. These include both initial emergency management and long‐term definitive care.

### 4.3 Resource Matrix: Necessary Elements of Trauma Care

On the vertical axis of each matrix are listed the specific elements of trauma care that are needed. These are divided into two categories: (1) knowledge and skills and (2) equipment and supplies. See Table 1 as an example.

Knowledge and skills imply that the staff (medical, nursing and others) have the requisite training to perform such diagnostic and therapeutic activities safely and successfully. This implies not only the requisite training in their basic education (school and postgraduate training), but also continuing education to maintain these skills. Requirements for continuing education (continuing professional development) are addressed in Table 15. In some situations, provision of services may require task sharing, with providers performing tasks above their usual level of training. Details of the supplemental training needed in these situations is included, where relevant, in the tables.

Equipment and supplies imply that these items are available to all who need them, without consideration of ability to pay, especially in true life‐threatening emergencies. This implies not only having them physically present in the facility but having them readily available on an ongoing basis; where appropriate, 24 hours a day, 7 days a week. It thus implies that organizational and administrative mechanisms exist to quickly replace depleted or expired stocks of supplies and medications, and to quickly repair non‐functioning equipment. The quality control mechanisms necessary to assure such provision of supplies and to assure the quality of medical care provided are addressed in Table 15.

The EsTC resource matrix goes into depth on the simple, vital services and related equipment. This is especially so for the immediately life‐threatening injuries to be addressed in the initial evaluation and resuscitation, such as the management of airway, breathing and circulation (Sections 5.1, 5.2, 5.3). For more complicated services, such as operative care of head, torso or extremity injuries (Sections 5.4, 5.5, 5.6, 5.7, 5.9, 5.10, 5.11, 5.12, 5.13, 5.14), the EsTC resource matrices go into less detail. For most of these more complicated issues, the elements of care to be provided are listed as a general service (e.g., laparotomy for trauma), with a basic discussion of what broad skills and equipment need to be available, but without a detailed, separate delineation of the specific skills or physical materials needed. Details of operating theater instruments, equipment, supplies and infrastructure, and of anesthetic capabilities are beyond the scope of this publication. The availability of a clinical service in these *Guidelines* implies the expertise and physical materials to carry out that service successfully and safely. In this regard, the reader is also referred to WHO publications on broader surgical and anesthetic issues (https://www.who.int/teams/integrated‐health‐services/clinical‐services‐and‐systems/surgical‐care).

### 4.4 Resource Matrix: Range of Health Facilities

On the horizontal axis of each matrix are listed the range of health facilities. It is acknowledged that the division between different levels is somewhat artificial, with actual facilities representing a continuum rather than discrete categories. It is also acknowledged that the capabilities of each level vary significantly between different countries. Working within these constraints, the authors have devised the following categories.

Clinic: Typically an outpatient clinic, usually staffed by nurses or mid‐level providers (may be known as physicians assistants, clinical officers, or similar) and occasionally, especially in middle‐income countries, by doctors. Such clinics can see many injured patients, and occasionally seriously injured patients, especially in rural locations with limited pre‐hospital emergency medical services. In the original *EsTC*, smaller facilities (e.g., village health posts staffed by providers with a few months training) were considered also. However, in this update, we restrict recommendations to clinics staffed by formally‐trained providers such as nurses.

First‐level hospital: Smaller hospitals, usually staffed by general doctors. They occasionally have a small number of specialists, such as a general surgeon or emergency physician. Such hospitals may or may not have operating theater capabilities.

Second‐level (referral) hospital: Hospitals whose personnel usually include at least a general surgeon. Staff at such facilities may also include orthopedic surgeons, emergency physicians, and members of other subspecialties. Such facilities have operating theaters.

Tertiary hospital: This includes hospitals with a broad range of subspecialty services. Such facilities are usually, but not exclusively, teaching or university hospitals. They usually represent the highest level of care in a country or large political division within a country. There are notable differences in the capabilities of tertiary care hospitals worldwide. In some countries, surgical staff may be quite extensive in their range of subspecialties, and in others, more limited.

These *Guidelines* do not make any recommendations regarding the optimum population served by each level of facility. However, this is of great relevance to the accessibility of trauma care by the population of a country. These issues are addressed by broader planning activities and should be considered by those planning trauma services for their country or area. Likewise, it is recognized that the different levels of facility will play differing roles within overall trauma treatment in different countries. For example, facilities staffed by non‐doctors and hospitals staffed by general practitioners are likely to care for a greater percentage of all injured patients in low‐income countries, whereas specialist‐staffed hospitals and tertiary care hospitals are likely to care for a greater percentage of all injured patients in middle‐income countries.

### 4.5 Resource Matrix: Designation of Priorities

For each cell within the matrix, the authors recommend those resources (vertical axis) that should be available at a specific level of the health care system (horizontal axis). The priority of each item was given a designation according to the following criteria.

Core (Co): The designated item should be “assured at that level in all cases, even in environments where access to resources is most severely restricted, including countries whose ministries of health have less than US$10 per capita to spend on health” [1]. These items in general would be “very cost‐effective” and, in many cases, if not present, could be provided primarily through improvements in organization and planning, with a minimal increase in expenditure. This designation is similar in concept to the term “essential” used in the original (2004) *Guidelines for EsTC*.

Extended (Ex): The designated item “increases the probability of a successful outcome.” It also adds cost. Such items are “not likely to be affordable or practical for all facilities of a given level,” especially in environments with the lowest access to resources. Hence, they are not listed as core. However, they would be more likely to be provided in better resourced locations. In some cases, the physical item itself might be low cost, but the training and monitoring needed to utilize it safely might be more resource intensive (e.g., endotracheal intubation). This designation is similar in concept to the term “desirable” used in the original (2004) *Guidelines for EsTC*.

Not applicable (NA) at that level. For example, a large expensive piece of equipment that would only be used in a hospital and would not realistically be expected to be found in a clinic would be NA for clinic level. The original *Guidelines for EsTC* used the term “irrelevant” for this category.

### 4.6 Application of Priorities

In national trauma planning, the authors anticipate that many countries may very appropriately decide to convert some of the items in the extended category to core. The converse is not true. Downgrading core items should be rare.

Throughout these *Guidelines*, more detail is provided on the elements of care that are deemed core. *However, it should be emphasized that items designated as extended are also an integral part of these Guidelines.* They are considered somewhat less important or cost‐effective than core items, but are not to be ignored. Items designated as core are those which should be able to be assured to all injured patients arriving at all facilities of a given level in all countries, even those of lowest income. *Hence, the core items are applicable to all health care facilities, including the most basic of facilities of a given level in the countries of lowest income:* for example, small basic clinics; small first‐level hospitals without surgical capabilities; specialist hospitals with only a general surgeon; and tertiary care facilities with a limited range of subspecialties. *Hence, many of the items that are designated as extended may indeed be applicable to many of these facilities, such as those in middle‐income countries or those with high numbers of trauma patients in all countries.* This is particularly an issue in the case of clinics, for which there is tremendous variation worldwide. Using the “least common denominator” of a small clinic in a low‐income country or area, the major emphasis for this level in these *Guidelines* is on rapid, basic first aid. Many other therapeutic items are listed as extended (e.g., oxygen, most medications, and IV fluids), as these are not applicable to all clinics worldwide. In national planning that addresses the clinic level, many such items might reasonably be upgraded to core for larger clinics and especially for those that do see a large number of seriously injured patients.

By way of an example of how the terms “core” and “extended” are actually applied in the recommendations, we look at airway management (Table 1). At all levels of the health care system, it is deemed core that health care personnel know the signs of airway obstruction and are skilled in manual maneuvers to keep an airway patent. The probability of success in airway management is increased by the provision of specific equipment and the skills to utilize it properly and safely. This includes equipment and skills for basic airway management, including oral airway, suction and bag–valve–mask. These are deemed core at hospital‐level facilities. The probability of success of airway management is increased even further by the provision of equipment and skills for advanced airway management, including endotracheal intubation and cricothyroidotomy. These are deemed core at specialist‐staffed hospitals. At each level, the probability of success of airway management is increased. However, both the need for resources (both equipment and training) and the potential for harm are increased. In environments in which more resources are available, and/or in which specific facilities handle a greater than average volume of trauma, it may be appropriate to change the “extended” designation to “core” at some or all facilities of a given level. For example, in an area where some clinics frequently receive seriously injured cases (especially in areas with limited formal prehospital emergency medical services), it would be reasonable to designate airway equipment such as oral airways, suction, and bag‐valve‐masks as core for those clinics. Similarly, for first‐level hospitals that receive high volume of trauma patients, it would be reasonable to designate the human and physical resources needed for endotracheal intubation as core. In short, most of the items that are designated as extended at a given level of the health care system could be considered for designation as core based on that facility's location, patient profile, and disease burden, as well as the staffing and other capabilities available at the facility.

### 4.7 What Is New in the Updated *Guidelines*

In both 2004 and 2025, consensus was derived among globally‐representative stakeholders as to what trauma care resources were feasible to promote in almost all situations. Fortunately, many items that were considered as extended in 2004 are now considered as core. This is likely the result of several factors, including: (1) increased attention to trauma and emergency care; (2) improved economic standards globally with many previously low‐income countries now being lower‐middle‐income countries, with a consequent decrease in the percent of people living in low‐income countries; and (3) improvements in several items of technology and their cost (with lower costs for some items now possible due to their manufacture in LMICs). Several examples include: pulse oximetry is now deemed core at all levels of the health care system (whereas it had been not applicable at the clinic level and extended at hospital levels in 2004); mechanical ventilators are now core at second‐ and third‐level hospitals (whereas they had been extended at these levels in 2004); ultrasonography for diagnosis of intra‐abdominal bleeding is now core at second‐ and third‐level hospitals (whereas it had been extended at these levels in 2004); portable X‐ray is now core at all hospital levels (whereas it had been core only at tertiary hospitals in 2004). Several resources have been deleted, as the evidence base for their use and resultant practice patterns change, such as discontinuing of right heart catheter (pulmonary artery catheter) for monitoring of fluid status. Multiple changes have been made in the list of trauma related medications, based on corresponding changes in WHO's Model List of Essential Medicines.

Reference for Section 41
*World Bank Open Data: Domestic General Government Health Expenditure per Capita (Current US$)* (2026), https://data.worldbank.org/indicator/SH.XPD.GHED.PC.CD.

## 5 Guidelines for Essential Trauma Care

For each of the following 14 categories of trauma care, a summary resource matrix is provided, preceded by a brief explanation of the rationale used in determining which elements of care are to be considered core or extended. An explanation of the organization of the resource matrices is provided in Section 4. Table 15 addresses administrative functions and Section 5.16 addresses special considerations for the care of injured children.

### 5.1 Airway Management

Airway management is one of the key components of emergency care. Its primary objective is to diagnose an obstructed or potentially obstructed airway, to clear the obstruction and keep the airway patent. Few medical emergencies, short of a complete cardiopulmonary arrest, are more immediately life‐threatening than the loss of an adequate airway. Failure to adequately manage airway patency and ventilation has been identified as a major cause of preventable death in trauma [1, 2].

In the initial assessment and management of any critically ill patient, the airway, breathing and circulation (ABC) are the first steps. The response to any acutely ill or injured patient must be met using a systematic approach, with the airway being the first priority. If any abnormalities are detected, measures to intervene are instituted immediately. The skills to assess a patient for obstruction of the airway, to establish and maintain a patent airway, and to ensure adequate ventilation and oxygenation of the patient, are therefore core.

At all levels of the health care system, it is deemed core that health care personnel know the signs of airway obstruction and are skilled in manual maneuvers to keep an airway patent while maintaining cervical spine protection. The probability of success of airway management is increased by the provision of specific equipment and the skills to utilize it properly and safely. This includes equipment and skills for basic airway management, including oral or nasal airway, suction and bag–valve–mask. These are deemed core at hospital‐level facilities. The probability of success in airway management is further increased by the provision of equipment and skills for advanced airway management, including endotracheal intubation and cricothyroidotomy. These are deemed core at specialist‐staffed (second‐level and tertiary) hospitals. At each level, the probability of success in airway management is increased. However, both the need for resources (both equipment and training) and the potential for harm are increased. Environments in which more resources are available, and/or in which there is a greater than average trauma volume in specific facilities, may wish to change the “extended” designation to core at some or all facilities of a given level.

At whatever level of the health care system it is decided to provide advanced airway capabilities (e.g., endotracheal intubation, cricothyroidotomy, or tracheostomy, depending on the circumstances—see end of Section 5.1), several safety assurances should be in place. This includes the provision that the equipment is readily available in whatever area receives the injured patient (casualty ward or emergency unit). This is aided by having the necessary equipment in pre‐assembled packs (where appropriate and feasible), stocked in the emergency area. Safety assurance also mandates that staff performing the various procedures be adequately trained to perform them successfully, with an acceptable rate of complications. This includes both the training received in basic education (e.g., medical or nursing or other professional school) and whatever continuing education might be required to maintain the skills. Given the potential for harm (in the form of esophageal intubation) with advanced airway management, inexpensive materials to assist in the clinical assessment of endotracheal tube placement should be provided whenever endotracheal intubation is performed. This includes principally an esophageal detector device (either bulb or syringe). Finally, given this potential for harm associated with advanced airway maneuvers, a quality assurance mechanism should be in place to track adverse events such as esophageal intubations. Further details can be found in Section 5.15 on quality improvement.

By way of further explanation of some of the equipment listed in the table, suction is an extremely important component of airway management. It can be provided at a low cost by manual and foot pump devices. These should be considered core in any hospital. Powered (e.g., electric) suction is considered core at higher level hospitals. Even in these institutions, availability of manual/foot pump devices should remain in event of power outage. Likewise, a stiff suction tip (Yankauer or equivalent) is a core component of an adequate suction set up. The term “basic trauma pack” implies a kit with a few basic instruments and supplies, including a scalpel, clamps, scissors, gauze, suture, syringe and needles. These represent a component of the minimum of physical resources needed to perform certain smaller procedures in the casualty ward/emergency department setting. Such procedures include cricothyroidotomy in Table 1. They also include some procedures mentioned later, such as chest tube insertion in Table 2. The basic trauma pack is considered core at all hospital levels.

At the clinic level, assessment of airway compromise and manual maneuvers to keep an airway patent are core. The next tier of interventions: insertion of oral or nasal airway, use of suction (including stiff suction tip), and assisted ventilation using a bag‐valve‐mask are deemed extended, given the need for training in their use in often very basic circumstances. However, in clinics that do receive at least an occasional seriously injured patient (and especially those in more remote areas with limited prehospital emergency medical services access), it would be reasonable to make these capabilities core. The use of more advanced airway capabilities, such as endotracheal intubation, would generally be in only very exceptional clinic circumstances.

These *Guidelines* indicate the use of cricothyroidotomy when a surgical airway is indicated. In general, this is performed more quickly and safely than a tracheostomy, especially by non‐specialists [3]. If needed for a prolonged period, it is usually converted to a tracheostomy after a few days.

**TABLE 1:** Airway management.

	Facility level^a^			
Airway: Knowledge and skills	Clinic	First	Second	Tertiary
Assessment of airway compromise	Co	Co	Co	Co
Manual maneuvers (chin lift, jaw thrust, recovery position, etc.)	Co	Co	Co	Co
Insertion of oral or nasal airway	Ex	Co	Co	Co
Use of suction	Ex	Co	Co	Co
Assisted ventilation using bag–valve–mask	Ex	Co	Co	Co
Endotracheal intubation	Ex	Ex	Co	Co
Cricothyroidotomy (with or without tracheostomy)	Ex	Ex	Co	Co
Airway: Equipment and supplies				
Oral or nasal airway	Ex	Co	Co	Co
Suction device: At least manual (bulb) or foot pump	Ex	Co	Co	Co
Suction device: Powered: Electric/pneumatic	Ex	Ex	Co	Co
Suction tubing	Ex	Co	Co	Co
Yankauer or other stiff suction tip	Ex	Co	Co	Co
Laryngoscope	Ex	Ex	Co	Co
Endotracheal tube	Ex	Ex	Co	Co
Intubating bougie/stylet	Ex	Ex	Co	Co
Esophageal detector device	Ex	Ex	Co	Co
Bag–valve–mask	Ex	Co	Co	Co
Basic trauma pack^b^	Ex	Co	Co	Co
Magill forceps	Ex	Co	Co	Co
Capnography	NA	Ex	Co	Co
Laryngeal mask airway/supraglottic airway	Ex	Ex	Co	Co
Other advanced airway equipment (Appendix 2)	NA	Ex	Ex	Ex

^a^ Facility level implies: clinic, first‐level hospital, second‐level hospital, or tertiary hospital.

^b^ Basic trauma pack implies a kit with basic instruments and supplies, including a scalpel, clamps, scissors, gauze, suture, syringe, and needles. These represent a component of the minimum of physical resources needed to perform certain smaller procedures in the casualty ward/emergency department setting. Such procedures include cricothyroidotomy in Table 1. They also include some procedures mentioned later, such as chest tube insertion in Table 2.

References for Section 5.11

D. O.
Alao
, 
A. A.
Cevik
, 
F.
Al Shamsi
, et al., “Preventable Deaths in Hospitalized Trauma Patients,” World Journal of Surgery
48, no. 4 (2024): 863–870, 10.1002/wjs.12109.38381056
2

T. L.
Sanddal
, 
T. J.
Esposito
, 
J. R.
Whitney
, et al., “Analysis of Preventable Trauma Deaths and Opportunities for Trauma Care Improvement in Utah,” Journal of Trauma
70, no. 4 (2011): 970–977, 10.1097/TA.0b013e3181fec9ba.21206286
3
American College of Surgeons
, Advanced Trauma Life Support. Student Course Manual, 10th ed. (ACS, 2018).

### 5.2 Breathing—Management of Respiratory Distress

The ability to assess a patient for respiratory distress and adequacy of ventilation is core at all levels of the health care system. This applies both to those initially evaluating the patient and to those who are providing definitive care. The only resources required for this function are training and a stethoscope. If no other resources are available at the level in question, it is anticipated that respiratory distress would usually constitute grounds for referral to the next highest level of the system.

Capabilities for the administration of oxygen to trauma patients in respiratory distress are core at all hospital facilities. This would be useful at all levels of the health system. It is recognized that this is currently beyond the realm of feasibility for many clinics in low‐income countries and other low‐resource settings. However, facilities that receive a moderate volume of seriously injured patients (e.g., those located along busier roadways), especially in middle‐income settings, might realistically be supplied with oxygen. The capability for administration of oxygen implies both health care providers capable of understanding the indications for its use and equipment and supplies to administer it in a timely fashion to trauma patients in respiratory distress. In most cases, this implies that the physical resources are present in the area where acute trauma patients are initially received.

The recognition of tension pneumothorax, its primary treatment by needle (or “finger”) thoracostomy and definitive treatment by tube thoracostomy are core at all hospital‐level facilities. This implies sufficient training in the diagnosis of tension pneumothorax and in the safe performance of the relevant procedures. It also implies the ready availability of the needed materials, including a basic trauma pack, chest tubes and one‐way flow‐seal drainage.

At the clinic level, capabilities for the temporary relief of tension pneumothorax with a needle thoracostomy could be considered extended in settings with the possibility of rapid evacuation to a site of definitive treatment. This would usually imply the existence of a prehospital emergency medical services system.

Recognition of the presence of a sucking chest wound and the ability to apply a three‐way dressing for immediate treatment is deemed core at all levels.

Assessment of the adequacy of supplemental oxygen is based primarily on clinical examination and monitoring of oxygen saturation through pulse oximetry, both of which are deemed core at all facility levels. Supplemental laboratory measurements (arterial blood gas concentration) provide further useful information. However, its cost prevents it from being deemed core for all environments. It is deemed as extended at first‐level hospitals and core at higher level hospitals.

When ventilation is inadequate, it can be supported manually (e.g., self‐inflating bag–valve–mask) or mechanically (e.g., ventilator). The preceding section on the airway has outlined the requirements for the bag–valve–mask. Mechanical ventilators have considerable utility for the physiological support of seriously injured patients. They are listed as extended at first‐level hospitals and core at higher level hospitals. It is acknowledged that they are expensive. However, suitable ventilators are available from suppliers in many LMICs at a fraction (even a tenth) of the cost of those available from high‐income country suppliers. The use of mechanical ventilators implies not only that they are physically present, but also that mechanisms exist to assure continual functioning by rapid repair, and that personnel are trained in their use. This would include respiratory therapists or nursing or other staff with adequate training in the use and routine maintenance of ventilators. The use of ventilators also implies doctors and nurses with sufficient training to care for mechanically‐ventilated patients. The latter implies the assessment of oxygenation status, the ability to recognize and correct problems (e.g., endotracheal tube obstruction), and skills in routine maintenance of ventilated patients, such as sterile suctioning, physiotherapy and postural drainage to reduce the risk of pneumonia. WHO guidance has addressed treatment of respiratory insufficiency due to respiratory infections (such as COVID), including use of mechanical ventilation [1].

In general, in respiratory failure in trauma patients, invasive mechanical ventilation is used (i.e., delivered through an endotracheal tube). Noninvasive mechanical ventilation modalities (such as continuous positive airway pressure and bilevel positive airway pressure) have some use in select trauma patients, such as: (1) patients with impairments in oxygenation, but patent airways, adequate ventilation, stable hemodynamics, and alert mental status; (2) after weaning and extubation from invasive mechanical ventilation.

**TABLE 2:** Breathing—Management of respiratory distress.

	Facility level			
	Clinic	First	Second	Tertiary
Breathing: Knowledge and skills				
Assessment of respiratory distress and adequacy of ventilation	Co	Co	Co	Co
Administration of oxygen	Ex	Co	Co	Co
Needle thoracostomy	Ex	Co	Co	Co
Chest tube insertion	NA	Co	Co	Co
Three‐way dressing	Co	Co	Co	Co
Breathing: Equipment and supplies				
Stethoscope	Co	Co	Co	Co
Oxygen supply (cylinder, concentrator or other source)	Ex	Co	Co	Co
Nasal prongs, face mask, associated tubing	Ex	Co	Co	Co
Needle and syringe	Ex	Co	Co	Co
Chest tubes	NA	Co	Co	Co
One‐way flow‐seal drainage	NA	Co	Co	Co
Pulse oximetry	Co	Co	Co	Co
Arterial blood gas measurements	NA	Ex	Co	Co
Bag–valve–mask	Ex	Co	Co	Co
Mechanical ventilator	Co	Ex	Co	Co

Reference for Section 5.21
World Health Organization
, Clinical Care of Severe Acute Respiratory Infections – Tool Kit (World Health Organization, 2022), https://www.who.int/publications/i/item/clinical‐care‐of‐severe‐acute‐respiratory‐infections‐tool‐kit.

### 5.3 Circulation—Management of Shock

#### 5.3.1 Assessment of Shock and Control of External Hemorrhage

The ability to assess a patient for the presence of shock is core at all levels of the health care system. The only resources required for this function are a clock or watch with second hand, a stethoscope and blood pressure (BP) cuff, and the relevant training. Training should include visual/manual assessment of circulation, including pulse, capillary refill time and skin temperature. If no other resources are available at the level in question, it is anticipated that shock would usually constitute grounds for referral to the next highest level of the system.

Control of external hemorrhage through manual pressure and through the application of a pressure dressing is core at all levels of the health care system. The only resources required are training and sufficient gauze bandages. Training regarding indications for the use of arterial tourniquets in extreme situations, as well as understanding the potential complications and the need for removal under controlled circumstances within several hours, is core at all levels. Splinting of fractured extremities as a means of decreasing internal hemorrhage is core at all levels.

More advanced, externally applied hemorrhage control measures include wrapping for potential pelvic fractures and deep interfascial packing for complicated wounds, such as landmine and machete wounds. These are deemed extended at the clinic level and core at all hospital levels. In locations with high rates of landmine injuries, it would be reasonable to make deep interfascial packing core at the clinic level.

#### 5.3.2 Fluid Resuscitation

Capabilities for fluid resuscitation include the equipment, the fluids themselves and the skills to administer them, monitor the response (including accurately monitoring fluid intake and output) and treat potential complications. The insertion of peripheral intravenous (IV) lines, percutaneously and by cutdown, and the use of crystalloid are deemed core at all hospital levels. These are extended at the clinic level. These should be considered for upgrading to core at the clinic level in locations where such clinics do receive seriously injured patients and are at such a distance that evacuation to a higher level of care will entail a delay of several hours or more. The insertion of central lines (including the lines themselves and the expertise to insert them safely) is deemed core at the upper two hospital levels and extended at first‐level hospitals. In the setting of first‐level hospitals, given the potential for complications, the insertion of central lines should usually only be considered for emergency situations in which access cannot be achieved by any other means.

Intraosseous lines for children, especially for those under 5 years, are deemed core at all hospital levels. Formal intraosseous needles would be ideal, but the ability to establish intraosseous access using any suitable large‐bore metal needle (e.g., spinal needle) is acceptable.

Capabilities for blood transfusion are deemed core at all hospital levels. A formal blood bank is the usual option. However, if not available, capabilities for immediate donation and administration of fresh whole blood are acceptable. Such capabilities are also needed for the treatment of obstetric hemorrhage and severe anemia. Many first‐level hospitals need to provide transfusions for these indications as well as for trauma. In addition to blood banks and immediate donations, more rapid delivery from centralized blood banks is another option. Preliminary evidence on some options for this is promising, such as for drone delivery [1]. The *Guidelines for EsTC* does not give recommendations on the various options for assuring blood availability. A minority of first‐level hospitals might not be expected to have such capabilities. These would include smaller facilities in less remote areas, with easy access to referral centers. Such facilities might be considered to more closely represent the clinic level than the first‐level hospitals considered in these *Guidelines.*

Blood transfusion may encompass transfusion of whole blood (whether fresh or banked) or use of components, including packed red blood cells, fresh frozen (or lyophilized freezedried) plasma, and platelets. Use of any of these will be influenced by capabilities of the institution's blood bank and the policies and practices of the institution or country. Any time that any blood product is administered, there should be capabilities to assure its safety, including screening for HIV, hepatitis B and C, and other blood borne diseases, depending on the geographic area. The use of blood should follow WHO blood transfusion safety guidelines [2, 3]. The use of blood also implies that it is being ordered by a clinician who knows the indications for transfusion in a trauma patient and is capable of recognizing and treating the potential complications of transfusion, monitoring the patient's response to transfusion and other fluid resuscitation, and assessing the patient for continued bleeding and the need for surgical intervention.

#### 5.3.3 Monitoring

The capability for monitoring a patient in shock for response to fluid therapy is deemed core at all hospital levels. This includes an understanding of the stages of hemorrhagic shock. It also includes the following basic equipment: clock or watch with second hand, stethoscope, blood pressure cuff and urinary catheter with collection bag (or improvized equivalent). These are deemed core at all hospital levels. Electronic cardiac monitoring (and related electrocardiogram) adds utility. It is acknowledged that they are expensive. However, suitable units are available from suppliers in many LMICs at a fraction of the cost of those available from high‐income country suppliers. Similarly, defibrillators are deemed core at all hospital levels. There are several options for defibrillators, ranging from simple versions with manual controls to options with pacing and synchronized cardioversion capabilities. The latter are more versatile, but also are more costly and require more involved training and maintenance. All of the above monitoring capabilities are non‐invasive. Central venous pressure monitoring also adds utility, but also the potential for complications. Globally, there has been increased use of point of care ultrasound to assess central filling pressures (e.g., vena cava width). Assessment of central filling pressures (by either central venous pressure monitoring and/or point of care ultrasound) as an aid to management of shock is deemed core at higher level hospitals and extended at first‐level hospitals.

Laboratory tests assist in the assessment of the presence of shock, the degree of bleeding and response to resuscitation. Hematocrit or hemoglobin monitoring is deemed core at all facility levels. Measurements of electrolytes (sodium, potassium, chloride, bicarbonate), blood urea nitrogen, creatinine, and lactate are all considered core at all hospital levels. Measurement of arterial blood gases (also covered in Table 2) are deemed extended at first‐level hospitals and core at higher level hospitals.

#### 5.3.4 Other Considerations

Most of the above pertains to the most common cause of shock in a trauma patient, hemorrhagic shock. Other causes include cardiogenic shock, neurogenic (or spinal) shock and septic shock. The ability to recognize these other causes of shock is deemed core at all hospital levels. Treatment of several of these causes of shock (especially cardiogenic and neurogenic) can involve use of vasoactive agents (e.g., pressors, inotropes, or similar medications, depending on the circumstances). Use of vasoactive agents is usually performed by continuous IV drip. This is deemed core at tertiary hospitals. It is deemed extended at first‐ and second‐level hospitals because of the cost of the infusion equipment and the need to adequately train both medical and nursing staff in the safe administration of these medications.

The ability to treat septic shock with antibiotics, fluid resuscitation and other supportive care is deemed core at all hospital levels. Appropriate treatment of sources of infection and underlying injury is covered further in several of the following sections.

Many patients in shock develop an ileus and are at risk of vomiting and aspiration of gastric contents. Hence, the availability of nasogastric (NG) tubes and the skills to insert them are deemed core at all hospital levels.

Hypothermia is a frequent complication of shock. The ability to recognize this and to treat it with external rewarming is deemed core at all levels. Capabilities to provide warmed fluids and gases, as well as other means of rewarming the body core, such as lavage via urinary catheters, nasogastric tube or intraperitoneal catheter, are considered extended at first‐level hospitals and core at higher level hospitals.

Weighing scales for children, in order to more accurately calculate fluid requirements, are considered core at all hospital levels and extended at the basic level.

**TABLE 3A:** Circulation and shock: Knowledge and skills.

	Facility level			
	Clinic	First	Second	Tertiary
Assessment and external control of haemorrhage				
Assessment of shock	Co	Co	Co	Co
Compression for control of haemorrhage	Co	Co	Co	Co
Arterial tourniquet in extreme situations	Co	Co	Co	Co
Splinting of fractures for haemorrhage control	Co	Co	Co	Co
Deep interfascial packing for severe wounds (e.g., landmine)	Ex	Co	Co	Co
Pelvic wrap for haemorrhage control	Ex	Co	Co	Co
Fluid resuscitation				
Knowledge of fluid resuscitation	Ex	Co	Co	Co
Peripheral percutaneous intravenous access	Ex	Co	Co	Co
Peripheral cutdown access	Ex	Co	Co	Co
Central venous access for fluid administration	NA	Ex	Co	Co
Intraosseous access for children under 5 years	Ex	Ex	Co	Co
Transfusion knowledge and skills	NA	Co	Co	Co
Monitoring				
Knowledge of resuscitation parameters	Ex	Co	Co	Co
More advanced monitoring (central venous pressure and/or point of care ultrasound)	NA	Ex	Co	Co
Other				
Differential diagnosis of causes of shock	Ex	Co	Co	Co
Use of vasoactive medications	NA	Ex	Ex	Co
Use of fluids and antibiotics for septic shock	NA	Co	Co	Co
Recognition of hypothermia	Co	Co	Co	Co
External rewarming in hypothermia	Co	Co	Co	Co
Use of warmed fluids	NA	Ex	Co	Co
Knowledge of core rewarming	NA	Ex	Co	Co

**TABLE 3B:** Circulation and shock: Equipment and supplies.

	Facility level			
	Clinic	First	Second	Tertiary
Assessment and external control of haemorrhage				
Clock or watch with second hand	Co	Co	Co	Co
Stethoscope	Co	Co	Co	Co
Blood pressure (BP) cuff	Co	Co	Co	Co
Gauze and bandages	Co	Co	Co	Co
Arterial tourniquet in extreme situations	Co	Co	Co	Co
Fluid resuscitation				
Crystalloid	Ex	Co	Co	Co
Blood transfusion capabilities	NA	Co	Co	Co
Intravenous infusion set (lines and cannulas)	Ex	Co	Co	Co
Intraosseous needle or equivalent	Ex	Ex	Co	Co
Central venous lines	NA	Ex	Co	Co
Monitoring				
Stethoscope	Co	Co	Co	Co
Blood pressure (BP) cuff	Co	Co	Co	Co
Urinary catheter	Ex	Co	Co	Co
Electrocardiogram (ECG)	NA	Co	Co	Co
Electronic cardiac monitoring	NA	Co	Co	Co
Defibrillator	Ex	Co	Co	Co
More advanced monitoring (central venous pressure and/or point of care ultrasound)	NA	Ex	Co	Co
Laboratory facilities for haemoglobin or haematocrit	Co	Co	Co	Co
Laboratory facilities for electrolytes and lactate	NA	Co	Co	Co
Laboratory facilities for arterial blood gases	NA	Ex	Co	Co
Other				
Vasoactive medications	NA	Ex	Ex	Co
Nasogastric (NG) tube	Ex	Co	Co	Co
Thermometer	Co	Co	Co	Co
Blankets for external rewarming	Co	Co	Co	Co
External rewarming using warmed blankets, electric heating blanket or other similar means	NA	Co	Co	Co
Warmers for IV fluids and other methods of rewarming body core	NA	Ex	Co	Co
Weighing scale for children	Ex	Co	Co	Co

References for Section 5.31

N. P.
Raykar
, 
V.
Raguveer
, 
Y. E.
Abdella
, et al., “Innovative Blood Transfusion Strategies to Address Global Blood Deserts: A Consensus Statement From the Blood Delivery via Emerging Strategies for Emergency Remote Transfusion (Blood DESERT) Coalition,” Lancet Global Health
12, no. 3 (2024): e522–e529, 10.1016/S2214-109X(23)00564-8.38365422
PMC108822072
World Health Organization
, Global Status Report on Blood Safety and Availability 2021 (World Health Organization, 2022).3
World Health Organization
, Guidance on Implementation of a Quality System in Blood Establishments (World Health Organization, 2023).

### 5.4 Management of Head Injury

Sections 5.1, 5.2, 5.3 describe in detail the specific skills, equipment and supplies needed to treat the immediately life‐threatening injuries addressed in the initial evaluation and management. For more complicated issues, these *Guidelines* provide less specific detail. Details of operating theater equipment and supplies are beyond the scope of this publication [1]. Hence, only a few exceptionally critical items are mentioned. Instead, the *Guidelines* state the services that should be provided, with the implication that the training, equipment and supplies needed to provide these services successfully and safely are present. Hence, the following Sections 5.4, 5.5, 5.6, 5.7, 5.9, 5.10, 5.11, 5.12, 5.13, 5.14 of the *Guidelines* list clinical services without division into skills and knowledge versus supplies and equipment.

Head injury (traumatic brain injury) is one of the major causes of trauma‐related death and disability worldwide. The assessment of neurological status, including determination of level of consciousness using the Glasgow coma scale, recognition of lateralizing signs, and determination of pupillary size and reflexes, are considered core at all levels of the health care system, in all countries. This requires only training and perhaps a source of artificial light such as a pocket torch. One of the most significant therapeutic modalities that needs to be applied broadly worldwide is the minimization of secondary brain injury through the maintenance of cerebral perfusion and oxygenation. Much of the mortality from traumatic brain injury is associated with secondary brain injury resulting from hypoxia and hypotension [2]. This reinforces the primary importance of the ABC outlined in Sections 5.1, 5.2, 5.3 above. Recognition of the importance of these factors in patients with head injuries is deemed core at all hospital levels.

A refinement on the above is to offset the propensity toward raised intracranial pressure (ICP) by avoiding overhydration, principally in hemodynamically stable patients. This knowledge and the understanding that head‐injured patients with hypovolemia also require appropriate hydration to prevent hypotension is deemed core at all hospital levels. This also includes use of elevation of the head of the bed to 30°, which is deemed core at all hospital levels. Ongoing treatment of raised ICP through such means as sedation, hyperosmolar therapy (such as with mannitol or hypertonic saline), paralysis, and cerebrospinal fluid drainage are deemed core at the tertiary care level. They are extended at the second‐level hospital if a neurosurgeon or other practitioner (e.g., intensivist, general surgeon) with the relevant training is present. Monitoring of ICP may aid in this management. Use of ICP monitoring is widespread, but has been the subject of considerable debate. It may be a useful adjunct to assist with management of raised ICP, but has not been conclusively shown to improve outcomes (as opposed to treatment of raised ICP based on clinical signs). Hence, the *Guidelines for EsTC* do not make recommendations regarding resource requirements for invasive ICP monitoring [3, 4, 5, 6, 7]. Similarly, the *Guidelines* do not make recommendations regarding resource requirements for non‐invasive ICP monitoring (e.g., transcranial Doppler with wave form analysis, automated pupillometry, and ultrasonographic optic nerve sheath diameter measurement). This topic has received increasing attention as a potential method to increase availability of ICP monitoring in LMICs [8]. Ongoing treatment of raised ICP in the intensive care unit setting is to be distinguished from one‐time boluses of mannitol or hypertonic saline, such as in the emergency unit setting. This covered further in the medications section (Table 12).

Intracranial mass lesions with pressure effect account for only around 10%–20% of comatose patients. However, timely decompression of these lesions significantly improves outcome. Treatment of these lesions is greatly facilitated by the availability of computerized tomography (CT). This is deemed core at tertiary hospitals and extended at all other hospital levels. Its high cost prevents it from being considered core at non‐tertiary hospitals. CT scans are indeed available in many locations, including low‐income countries, but many factors preclude their ready availability to all patients with suspected intracranial mass lesions. These include cost, and in some cases associated mandatory fees, as well as prolonged periods of breakdown. Some countries may decide to make CT scanning core at non‐tertiary hospitals in their own plans. This requires not only the physical presence of the machine, but also timely 24‐hour availability to all severely head‐injured patients, without regard to ability to pay. It also includes facilities for maintenance and rapid repair within 24 hours. In addition, basic quality improvement programs should assure that all patients warranting CT scan of the head (generally Glasgow coma scale of 8 or less) are promptly scanned (generally within 2 hours of arrival to the hospital).

Surgical treatment of intracranial mass lesions is classified as basic (burr hole) or advanced (including craniotomy, craniectomy, treatment of intracerebral hematoma, etc.). Relief of raised ICP from intracranial mass lesions by burr hole alone implies the skill to perform the operation and the drills or other suitable equipment needed. Ideally any of the above‐noted procedures should be performed by a neurosurgeon, who are usually found only at tertiary hospitals. At these tertiary hospitals, burr holes should be considered core. Some first‐level and second‐level hospitals are situated in isolated places with minimal capabilities for timely referral. In these locations, burr holes may reasonably be considered to be extended. This would imply that a general surgeon (or other provider) with suitable surgical experience would be authorized to perform them. Some tertiary care hospitals in LMICs do not have neurosurgeons. At these facilities, the capability to perform burr holes should still be core.

The more advanced procedures noted above should be available at tertiary facilities when a neurosurgeon is available. The lack of neurosurgeons at some tertiary facilities (or lack of 24 hour coverage by neurosurgeons) leads to these *Guidelines* deeming these advanced procedures as extended at tertiary facilities.

A particular subset of neurosurgical procedures, elevation of depressed skull fractures (both open and closed), parallels the resource availability of burr holes and has the same designations: extended at first‐ and second‐level hospitals and core at tertiary.

Finally, malnutrition in patients with traumatic brain injury has been associated with worsened outcome [3]. Maintenance of at least baseline caloric and protein requirements should be assured, including nasogastric feeding if the patient is comatose. This is deemed core for all head‐injured patients with altered neurological status at all hospital levels. This also applies to patients with a prolonged inability to eat, whether for head injury or other forms of trauma.

While the *Guidelines for EsTC* do not go into detail on specific procedures, given the complexity of traumatic brain injury management it is recommended, within the limitations of each institution's capability, that a traumatic brain injury treatment guideline is followed and should be derived from international best‐practice recommendations currently available.

**TABLE 4:** Head injury.

Resources	Facility level			
	Clinic	First	Second	Tertiary
Recognize altered consciousness; lateralizing signs, pupils	Co	Co	Co	Co
Maintain normotension and oxygenation to prevent secondary brain injury	Ex	Co	Co	Co
Avoid overhydration in the presence of raised ICP (with normal BP)	Ex	Co	Co	Co
Elevation of head of bed to 30°	Ex	Co	Co	Co
Treatment of raised ICP	NA	NA	Ex	Co
CT scans	NA	Ex	Ex	Co
Burr holes (skill plus drill or other suitable equipment)	NA	Ex	Ex	Co
More advanced neurosurgical procedures	NA	NA	Ex	Ex
Surgical treatment of depressed skull fractures	NA	Ex	Ex	Co
Maintenance of requirements for protein and calories	NA	Co	Co	Co

Abbreviations: CT, computerized axial tomography; ICP, intracranial pressure.

References for Section 5.41
World Health Organization
, 
WHO Trauma & Emergency Surgery Kit (WHO TESK)
 (2026), https://cdn.who.int/media/docs/default‐source/documents/emergencies/tesk‐kit‐information‐note‐21‐june‐2019.pdf?sfvrsn=5e3ad157_2.2

L.
Yang
, 
C. F.
Opalak
, and 
A. B.
Valadka
, “Chapter 22: Brain,” in Trauma, ed. 

D. V.
Feliciano

, 

K. L.
Mattox

, and 

E. E.
Moore

, 9th ed. (McGraw‐Hill Medical, 2021).3
Brain Trauma Foundation
, 
Guidelines for the Management of Severe TBI
, 4th ed. (2016). https://braintrauma.org/coma/guidelines/severe‐tbi.4
Brain Trauma Foundation
, Guidelines for the Management of Pediatric Severe TBI, 3rd ed. (Brain Trauma Foundation, 2019). https://braintrauma.org/coma/guidelines/pediatric.5

A. M.
Cook
, 
G.
Morgan Jones
, 
G. W. J.
Hawryluk
, et al., “Guidelines for the Acute Treatment of Cerebral Edema in Neurocritical Care Patients,” Neurocritical Care
32 (2020): 647–666, 10.1007/s12028-020-00959-7.32227294
PMC72724876

N.
Stocchetti
, 
E.
Picetti
, 
M.
Berardino
, et al., “Clinical Applications of Intracranial Pressure Monitoring in Traumatic Brain Injury: Report of the Milan Consensus Conference,” Acta Neurochirurgica
156, no. 8 (2014): 1615–1622, 10.1007/s00701-014-2127-4.24849391
7

T.
Zoerle
, 
E.
Beqiri
, 
C. A. I.
Åkerlund
, et al., “Intracranial Pressure Monitoring in Adult Patients With Traumatic Brain Injury: Challenges and Innovations,” Lancet Neurology
23, no. 9 (2024): 938–950, 10.1016/S1474-4422(24)00235-7.39152029
8

C.
Robba
, 
E.
Picetti
, 
S.
Vásquez‐García
, et al., “The Brussels Consensus for Non‐Invasive ICP Monitoring When Invasive Systems Are Not Available in the Care of TBI Patients (The B‐ICONIC Consensus, Recommendations, and Management Algorithm),” Intensive Care Medicine
51, no. 1 (2025): 4–20, 10.1007/s00134-024-07756-2.39847066


### 5.5 Management of Neck Injury

This section will deal primarily with penetrating neck trauma. Blunt trauma causing spinal injury is included in the section on the spine. All forms and causes of airway obstruction are covered in Section 5.1.

The ability to recognize platysmal penetration is deemed core at all hospital levels. This implies the ability to recognize the physical finding, understand its significance and either treat the patient accordingly or refer to the next level of the health care system. Such skills are extended for the clinic level, but might be considered as core in areas with high rates of penetrating trauma.

The ability to perform external control of hemorrhage is core at all levels of the health care system. More advanced initial maneuvers include packing and balloon catheter tamponade. These are deemed extended at first‐level hospitals and core at higher level hospitals. These imply the requisite skills in airway management (Section 5.1), especially as compression of neck wounds may exacerbate airway compromise. Such packing, with or without balloon tamponade, implies a level of skill that would not be expected, and hence would be extended at clinics. However, it might be useful even at clinics in areas with high levels of penetrating trauma. Ancillary diagnostic tests include contrast radiography (oesophagography), endoscopy (laryngoscopy, bronchoscopy) and angiography. The high cost of the latter prevents it from being considered core.

Surgical exploration of penetrating neck trauma is the definitive diagnostic test and the definitive mode of treatment. It is deemed core at second‐level and tertiary hospitals. In more remote, rural low‐income areas, it is considered as extended at first‐level hospitals, primarily in those locations where facilities for referral are limited. In such cases, appropriate training in exploration, repair of esophageal injuries and primary suturing of vascular injuries should be assured for any surgeon or other doctor expected to undertake such work.

**TABLE 5:** Penetrating neck injury.

Resources	Facility level			
	Clinic	First	Second	Tertiary
Recognize platysmal penetration	Ex	Co	Co	Co
External pressure for bleeding	Co	Co	Co	Co
Packing, balloon tamponade for bleeding	Ex	Ex	Co	Co
Contrast radiography, endoscopy	NA	NA	Ex	Co
Angiography	NA	NA	Ex	Ex
Surgical skills to explore neck	NA	Ex	Co	Co

### 5.6 Management of Chest Injury

Core items for the care of immediately life‐threatening chest injuries have been addressed in Section 5.2. These include capabilities for the emergency insertion of a chest tube, oxygenation and respiratory support. An extension of such issues is the ability to collect blood from chest tube output for autotransfusion. This is core at second‐level and tertiary hospitals. It is considered extended at first‐level hospitals, given the cost, need for sterility, and possibly infrequent use at this level.

Most chest injuries, whether blunt or penetrating, are managed without surgical operation. Major preventable complications are atelectasis and pneumonia. The prevention of these is contingent on adequate pulmonary toilet, which is in turn contingent on adequate pain control. These are all low‐cost capabilities and should be core at all hospital levels. Pain control implies an adequate supply of analgesics, which is addressed in Section 5.12. In addition to the physical availability of the medications, adequate pain control implies the skills needed to understand the importance of pain control in a patient with a chest injury, the ability to assess a patient for such pain and its effect on their respiratory status, and the ability to assess adequate response to analgesia. Such skills are deemed core at all hospital levels.

Useful adjuncts include regional anesthesia, such as rib blocks (e.g., intercostal nerve blocks) and epidural analgesia. These would imply the availability of long‐lasting local anesthetics (e.g., bupivacaine). They also imply training to be able to perform the blocks satisfactorily and safely, and to recognize and treat potential complications. Capabilities for rib blocks are core at second‐level and tertiary level. They are extended at first‐level hospitals, if these are in more remote locations with limited capabilities for referral. Epidural analgesia would usually only be available where a fully trained physician anesthetist is available. Due to this restriction and the cost of the special catheters needed, this capability is deemed core only at tertiary and extended only for second level hospitals. Other similar methods of pain treatment include paravertebral or erector spinal blocks, among others.

Surgery for chest injuries can be classified as intermediate (including ligation of chest wall bleeding, pulmonary tractotomy and pulmonary resection) or advanced (including aortic repair with prosthetic graft). Intermediate thoracotomy capabilities are deemed extended at second‐level and core at the tertiary level. Given the level of skill needed for such procedures and need for post‐operative intensive care, they would not be deemed extended at first‐level hospitals, except under the most extreme circumstances. Performance of these procedures at second‐level hospitals would imply the presence of a surgeon with the requisite skill, and adequate operative and postoperative facilities. The balance between these capabilities and the capability for rapid transfer to tertiary facilities needs to be determined on a local basis. Advanced thoracic surgical capabilities are deemed extended at the tertiary care level, because of the high cost and hence low availability of more advanced materials, such as aortic grafts.

Any hospital performing thoracic surgical procedures should have basic quality improvement mechanisms in place to track the outcomes of such procedures.

**TABLE 6:** Chest injury.

Resources	Facility level			
	Clinic	First	Second	Tertiary
Autotransfusion from chest tubes	NA	Ex	Co	Co
Adequate pain control for chest injuries/rib fractures	Ex	Co	Co	Co
Respiratory therapy for chest injuries/rib fractures	NA	Co	Co	Co
Rib block or intrapleural block	NA	Ex	Co	Co
Epidural analgesia (or equivalent)	NA	NA	Ex	Co
Skills and equipment for intermediate thoracotomy	NA	NA	Ex	Co
Skills and equipment for advanced thoracotomy	NA	NA	NA	Ex

### 5.7 Management of Abdominal Injury

The capability to utilize basic physical examination to assess an injured patient for the possibility of intra‐abdominal injury requiring surgical treatment or other intervention is deemed core at all levels of the health care system. Also needed are the skills and equipment (BP cuff and stethoscope) to assess the patient for shock.

Such physical examination needs supplementation with ancillary diagnostic tests in equivocal cases and when the patient's abdominal examination is unreliable due to altered mental status. This is usually fulfilled by ultrasound, CT scan and diagnostic peritoneal lavage (DPL). After physical examination, ultrasound would generally be the next diagnostic modality to be considered, given that it is non‐invasive (vs. DPL) and less costly and usually faster than a CT. Ultrasound examination of the abdomen in trauma is usually performed by focused assessment with sonography for trauma (FAST) and is used to detect the presence of haemoperitoneum. Given the widespread availability of ultrasound for other purposes (e.g., obstetrics), its moderate cost, and its non‐invasiveness, it is considered: extended at first‐level hospitals and core at higher level hospitals. For first‐level hospitals with high volumes of trauma, it would be reasonable to designate ultrasound as core. When utilized, it should be recognized that the skills needed to perform US examination for haemoperitoneum are different and somewhat more advanced than those needed to perform basic obstetric evaluation, which is available in many LMIC environments. When designated core for the evaluation of abdominal trauma, the following need to be assured: 24‐hour availability of the equipment (which implies timely repair of any malfunctioning equipment); 24‐hour availability of staff skilled in the performance of the procedure; and ongoing monitoring of the accuracy of the results of the scans.

CT scanning adds utility in the evaluation of the injured abdomen, especially as regards the retroperitoneal structures. It is deemed: extended at first‐level and second‐level hospitals and core at tertiary hospitals. Its cost prevents it from being deemed core more widely. When designated core for the evaluation of abdominal trauma in a national plan, the same caveats apply as for the use of CT for head trauma: prompt availability without regard to ability to pay; maintenance and timely repair; and quality assurance monitoring.

DPL can supplement the above diagnostic modalities and can be used in their place if they (FAST, CT) are not available. The capability to perform DPL implies provision of the fluid and the inexpensive equipment involved, as well as the skills needed to perform the procedure safely. Such capability is deemed core at hospitals at second‐level and tertiary care levels. It is extended at first‐level hospitals. It might especially need to be considered for first‐level hospitals with high trauma volumes. The need to assure adequate and safe performance of the procedure will often mandate continuing education and periodic practice, especially in circumstances of low trauma volumes, where the procedure is only infrequently utilized. These requirements prevent DPL from being considered core at all first‐level hospitals. It should also be noted that the simple first step of a DPL is peritoneal aspirate (without lavage). With large haemoperitoneum, a positive aspirate is often sufficient confirmation to decide on the need for laparotomy.

The capability to perform a trauma laparotomy and to deal with the wide range of potential injuries to the intraperitoneal and retroperitoneal structures is one of the mainstays of definitive care of the seriously injured patient and is deemed core for second‐level and tertiary care hospitals. This is primarily wherever fully trained general surgeons are available. As with neurosurgical and thoracic trauma operations, abdominal trauma operations can be roughly categorized into intermediate and advanced. Intermediate implies procedures such as exploration, recognition of injured structures, hemostasis through packing, splenectomy, hepatic packing and suturing, repair of perforated bowel, and bowel resection and anastomosis. Advanced implies procedures in the retroperitoneum, hepatic resection and other more difficult procedures.

The capability to perform intermediate trauma laparotomy is extended for first‐level hospitals and might especially need to be provided in rural, low‐income settings, where general practitioners (when fully trained general surgeons are not available) are called upon to perform a wide range of basic‐to intermediate‐level abdominal surgical procedures, such as Caesarean section, salpingectomy for ruptured ectopic pregnancy, plication of typhoid ileal perforation, and bowel resection for strangulated hernia. In such circumstances, trauma‐related procedures that are often required include those intermediate‐level procedures listed above. In some circumstances, they may include damage‐control laparotomy prior to transfer to higher‐level hospitals. Whenever general practitioners are called upon to perform aspects of trauma laparotomy, the skills needed to perform such procedures effectively and safely should be assured during basic medical school education and by continuing education courses.

At whatever facility trauma laparotomy of either intermediate or advanced level is performed on a routine basis, the quality of the procedures should be monitored and assured by some form of quality improvement programme. This would look at such aspects of care as missed injuries, delays in performance of emergency laparotomy and reoperation rates.

In prior section (on chest injury), autotransfusion from chest tubes was deemed extended at first‐level hospitals and core at second‐level and tertiary. Autotransfusion from abdominal bleeding in trauma, especially vascular injury, is performed in many high‐income countries, but the technology needed to do it is more costly than that for chest injury. There are also contravening issues such as potential for contamination from bowel injury. Two recent reviews on the topic of autotransfusion/intraoperative autotransfusion have not conclusively decided in favor of recommending these modalities globally at this time [1, 2]. One review did note that early research on intraoperative recovery and subsequent autotransfusion using a low‐cost globally applicable device was “promising” as regards safety. Intraoperative transfusion from abdominal trauma may eventually prove to be cost‐effective and recommendable. However, at the current time, given the preliminary information available, these *Guidelines* defer recommendation on them.

**TABLE 7:** Abdominal injury.

Resources	Facility level			
	Clinic	First	Second	Tertiary
Clinical assessment	Co	Co	Co	Co
Diagnostic peritoneal lavage (DPL)	NA	Ex	Co	Co
Ultrasonography (FAST)	NA	Ex	Co	Co
CT scan	NA	Ex	Ex	Co
Skills and equipment for intermediate laparotomy	NA	Ex	Co	Co
Skills and equipment for advanced laparotomy	NA	NA	Co	Co

Abbreviations: CT, computerized axial tomography; FAST, focused assessment with sonography for trauma.

References for Section 5.71

N. P.
Raykar
, 
V.
Raguveer
, 
Y. E.
Abdella
, et al., “Innovative Blood Transfusion Strategies to Address Global Blood Deserts: A Consensus Statement From the Blood Delivery via Emerging Strategies for Emergency Remote Transfusion (Blood DESERT) Coalition,” Lancet Global Health
12, no. 3 (2024): e522–e529, 10.1016/S2214-109X(23)00564-8.38365422
PMC108822072

M.
Palmqvist
, 
J.
Von Schreeb
, and 
A.
Älgå
, “Autotransfusion in Low‐Resource Settings: A Scoping Review,” BMJ Open
12, no. 5 (2022): e056018, 10.1136/bmjopen-2021-056018.PMC911500635577473

### 5.8 Management of Extremity Injury

Injuries to the extremities are the primary cause of injury‐related disability in many countries. These disabilities can be greatly reduced if promptly recognized and corrected. Functional disabilities due to neglected or late treatment of these injuries continue to constitute a major burden on the developing world. The ability of individuals to return to work may be compromised and they may thus become a burden on their families and communities.

The recognition of major limb injuries and associated neurovascular compromise (including compartment syndrome) are core at all health care levels. The skills and resources required to immobilize limb injuries are considered core at all levels, including clinics, as appropriate immobilization can reduce or stop hemorrhage, provide pain relief, correct deformities and ensure safe transport. In unstable injuries, particularly those involving the cervical and thoracolumbar spine, immobilization may also limit secondary neurological damage, as discussed in Section 5.9 (Management of spinal injury).

It is recognized that there will be great variation in types of immobilization devices used across various countries. There will be indigenous material and designs for devices and various types of splints used to immobilize the injured extremity. Any improvisation that is inexpensive and based on scientific principles should be encouraged. Individual countries may seek professional expertise as they standardize their immobilization devices to ensure patient safety. Quite often, such devices are used as a part of definitive treatment in many conditions. All health workers caring for injured patients are expected to have the skills to provide suitable immobilization to the injured patient, as transportation may be necessary for definitive care. It is core that necessary immobilization devices for major extremity injuries, including hand injuries, be made available at the clinic level.

Spine boards (or equivalent methods of immobilizing the spine) (see Section 5.9 for further details), which can be produced at low cost, are considered to be extended equipment at the clinic level but will be core at higher levels of care. Immobilization technique and resources for wrapping pelvic fractures are deemed core even at the clinic as this can be performed with a piece of cloth and may save many lives by minimizing blood loss in unstable fractures of the pelvis. Similarly, splinting fractures of the femoral shaft will be helpful in reducing blood loss and providing pain relief, particularly in settings where transportation times are long.

A spectrum of procedures is required for definitive management of fractures, both those presenting acutely and those with delayed presentation. These include closed manipulation and casting, skeletal traction, external fixation (and its functional equivalent, pins and plaster), internal fixation, and irrigation and debridement (toileting) of complex extremity wounds, including open fractures. All of these are core at tertiary hospitals, which in general have orthopedic surgeons. All of these are core at second‐level hospitals. Depending on the country or area involved and its resources, second‐level hospitals may have orthopedic surgeons. If they do, all of the above are pertinent. If they do not, general surgeons with appropriate training and experience may be required to perform the above‐mentioned procedures, especially in circumstances where capabilities for referral to tertiary facilities are limited. The increased infection potential with more aggressive procedures, such as internal fixation, must be kept in mind. Hence, in addition to the physical presence of the implants and equipment, and to the skills necessary to conduct the procedures, a sufficiently sterile operating theater environment is core whenever internal fixation itself is considered core.

In some rural, low‐income circumstances, the above spectrum of capabilities might be possibly required for first‐level hospitals, and hence are deemed extended at this level. This would include some procedures carried out for definitive care. It might also include procedures such as irrigation and debridement of open fractures in circumstances where transfer to higher levels is possible but often delayed for several days.

A variety of other procedures need to be considered in the armamentarium of care of extremity injuries. These include management of injured hands, tendon lacerations and compartment syndrome. They also include an understanding of the indications for amputation and the capabilities to perform this safely. In all cases in which practitioners are routinely called upon to provide a level of care that is above and beyond what they would ordinarily be considered to be trained for, their training should be maximized as part of planning for core trauma care services. This would include training in medical school and postgraduate programs, as well as continuing education. It would include training for general practitioners and general surgeons in the above‐mentioned spectrum of skills. In the case of general surgeons, it might also include training in higher‐level orthopedic care, such as internal fixation and measurement of compartment pressures, and plastic surgery procedures, such as flap coverage.

As with other topics considered in previous sections, the availability of a service implies the skills needed to provide it effectively and safely, as well as the needed equipment. The relevant equipment must not only be physically present, but also promptly available to all who urgently need it, without regard to ability to pay, and worn‐out or broken equipment must be repaired or replaced. As regards such physical resources, the care of injured extremities entails diagnostic (e.g., radiographic) equipment, implants and operative equipment, the latter of which will not be dealt with further in these *Guidelines*. X‐ray facilities are generally considered essential for the diagnosis, treatment and successful outcome of skeletal injuries. X‐rays are thus deemed core for all hospital levels and extended at the clinic level. Portable X‐rays assist in the management of patients in traction and during operative procedures. Capabilities for portable X‐rays should be core at all hospital levels. C‐arm image intensifier (fluoroscopy) is considered an integral part of the orthopedic armamentarium in many settings as it offers accuracy, reduces operative time, decreases radiation exposure, allows closed procedures and hence saves blood loss and reduces infection rate [1, 2]. As with other higher‐cost technology covered by this publication (e.g., ventilators), suitable image intensifiers are available from LMIC suppliers at a fraction of the cost of those from high‐income country supplies. Image intensification is considered as core at tertiary hospitals and extended at second level hospitals. It might be considered as core for second level hospitals if an orthopedic surgeon is present and/or if there is a high burden of extremity injuries managed at that hospital.

It is noted that in countries with poor access to resources, non‐operative treatment is often offered for fractures, despite the fact that operative repair would result in a better functional outcome. The reasons for this include the unavailability of implants, equipment and imaging capability, lack of surgical training, lack of a good operative environment or simply inability on the part of the patient to pay for such treatment. It is hoped that the benefits of early mobility through stabilization of fractures will eventually be passed on to every injured patient in whom internal/external fixation would be helpful to prevent disability.

The range of implants and equipment used for external and internal fixation varies greatly between countries and between hospitals in the same country. The choice of implants will depend primarily on the training and capability of the surgeons, and on the availability of implants and other resources. No attempt has been made to list the hardware required for fixation of fractures, but it is expected that a country will be able to standardize its own requirements (implants and equipment sets) with local professional expertise. Inexpensive metal implants are used in many countries, with varying degrees of success. Countries may address the quality assurance of metal implants through appropriate mechanisms, in due course.

Complications such as pressure sores may arise from prolonged immobilization. Hence, proper management of immobilized patients (e.g., log rolling, frequent turning and early removal of spine boards) is core at all hospital levels.

**TABLE 8:** Extremity injury.

Resources	Facility level			
	Clinic	First	Second	Tertiary
Recognition of neurovascular compromise; disability‐prone injuries	Co	Co	Co	Co
Basic immobilization (sling, splint)	Co	Co	Co	Co
Spine board	Ex	Co	Co	Co
Wrapping of pelvic fractures for hemorrhage control	Co	Co	Co	Co
Skin traction	NA	Ex	Co	Co
Closed reduction	Ex	Ex	Co	Co
Skeletal traction	NA	Ex	Co	Co
Operative wound management	NA	Ex	Co	Co
External fixation (or its functional equivalent: Pins and plaster)	NA	Ex	Co	Co
Internal fixation	NA	Ex	Co	Co
Tendon repair	NA	Ex	Co	Co
Hand injury: Assessment and basic splinting	Co	Co	Co	Co
Hands: Debride, fix	NA	Ex	Co	Co
Measurement of compartment pressures	NA	Ex	Ex	Co
Fasciotomy for compartment syndrome	NA	Ex	Co	Co
Amputation	NA	Ex	Co	Co
X‐ray	Ex	Co	Co	Co
Portable X‐ray	NA	Co	Co	Co
Image intensification (C‐arm)	NA	NA	Ex	Co
Proper management of immobilized patient to prevent complications	Ex	Co	Co	Co

References for Section 5.81

Y.
Chan
, 
L.
Banza
, 
C.
Martin
Jr.
, 
W. J.
Harrison
, “Essential Fracture and Orthopaedic Equipment Lists in Low Resource Settings: Consensus Derived by Survey of Experts in Africa,” BMJ Open
8, no. 9 (September 2018): e023473, 10.1136/bmjopen-2018-023473.PMC6144338302243992

I.
Ojodu
, 
A.
Ogunsemoyin
, 
S.
Hopp
, et al., “C‐Arm Fluoroscopy in Orthopaedic Surgical Practice,” European Journal of Orthopaedic Surgery & Traumatology
28, no. 8 (2018): 1563–1568, 10.1007/s00590-018-2234-7.29796825


### 5.9 Management of Spinal Injury

Recognition of the presence or risk of spinal injury is core at all levels of the health care system. The only resource needed for this is training. Included in this is the necessity to monitor neurological function at regular intervals, such as hourly, in the acute phase of injury, and this should be considered core for all levels of care.

It is increasingly recognized that patients with spinal cord injury, especially acute cervical spinal cord injury, may experience severe hypotension and severe problems in maintaining an airway and adequate ventilation. The risk of further neurological deterioration is increased when the ABC's of trauma management are neglected. Therefore, as with prevention of secondary brain injury, recognition of the importance of these factors in patients with spinal injury is deemed core at all hospital levels.

A holistic approach to the prevention of complications should be considered core at all hospital levels of care and during all phases of management, from the acute phase to the rehabilitation phase. The most common complications, which increase morbidity and mortality, are pressure sores, urinary retention, urinary infection, pneumonia, and deep venous thrombosis. To prevent pressure sores, patients should be log‐rolled every 2 hours. These items are also desirable (and hence deemed extended) at the clinic.

Care of patients with spinal cord injuries is aided by use of a standardized classification system, such as those encompassed by the International Standards for Neurological Classification of Spinal Cord Injury [1]. Although clinics and smaller hospitals would not be expected to use such a classification system, it should be core for all specialists caring for patients with injuries of the spinal cord in tertiary hospitals to use such a system.

It is anticipated that patients with spinal injuries or suspicion thereof would be rapidly referred to the next highest level in the health care system, where they could be more adequately managed, in terms of diagnosis and treatment. Ideally, patients should arrive at tertiary care centers within 2 hours of injury. Appropriate handling of patients, with the use of simple techniques such as log‐rolling and the avoidance of undue movement during transport, is likewise core at all levels of the health care system. Immobilization of the spine may be performed with a variety of devices, depending on the country, level of the health care system, and type of injury. These include: C‐collars, block/neck rolls, backboards, among others. Ability to immobilize the spine in some suitable manner is considered core at all levels of the health care system. This includes padding of backboards and early removal to prevent pressure sores.

With respect to diagnosis, plain X‐rays of the spine are often used. Plain X‐rays are addressed in more detail in Sections 5.8 (Management of extremity injury) and 5.13 (Diagnosis and monitoring). Computerized tomography (CT) and magnetic resonance imaging (MRI) have great utility in the management of patients with spinal injury. However, their high cost prevents them from being designated core, other than for CT at tertiary hospitals. When they are deemed core as part of a national plan, their continual functioning and availability on an emergency basis (24 hours a day, 7 days a week) should be considered as an integral part of core status.

A variety of spinal injuries may be managed non‐operatively. These include stable fractures with or without neurological injury. They also include some unstable bony and ligamentous injuries for which either surgical fixation or non‐operative management could be used. Adjuncts to such non‐operative therapy include bed rest, cervical spine braces, halo devices and cervical spine traction. Halo devices are especially useful in hospitals with limited surgical capability. The ability to manage selected spinal injuries non‐operatively includes the training to recognize which injuries are appropriate for such management, and the equipment to provide non‐operative management. Such capabilities are deemed core in second‐level and tertiary care hospitals. In more remote rural low‐income areas, such capabilities might be possibly required (and hence deemed extended) at first‐level hospitals.

Management of complicated spinal cord injuries as appropriate by surgical means should be core at tertiary care facilities. This would imply the presence of an orthopedic or neurological surgeon with appropriate training. In some cases, it would be possibly required (and hence deemed extended) at second‐level hospitals, if the availability of tertiary care facilities is limited and if the personnel with the necessary expertise are available.

As with head injury (above), given the complexity of spinal injury management it is recommended, within the limitations of each institution's capability, that a spinal injury treatment guideline is followed and should be derived from international best‐practice recommendations currently available.

**TABLE 9:** Spinal injury.

Resources	Facility level			
	Clinic	First	Second	Tertiary
Assessment—Recognition of presence or risk of spinal injury	Co	Co	Co	Co
Immobilization	Co	Co	Co	Co
Monitoring of neurological function	Co	Co	Co	Co
Assessment by a standardized classification system	NA	NA	Ex	Co
Maintain normotension and oxygenation to prevent secondary neurological injury	Ex	Co	Co	Co
Holistic approach to prevention of complications—Especially pressure sores and urinary retention/infection	Ex	Co	Co	Co
CT scan	NA	Ex	Ex	Co
MRI	NA	NA	Ex	Ex
Non‐surgical management of spinal injury (as indicated)	NA	Ex	Co	Co
Surgical treatment of spinal injury	NA	NA	Ex	Co
Surgical treatment of neurological deterioration in the presence of spinal cord compression	NA	NA	Ex	Co

Abbreviations: CT, computerized axial tomography; MRI, magnetic resonance imaging.

Reference for Section 5.91

R.
Rupp
, 
F.
Biering‐Sørensen
, 
S. P.
Burns
, et al., “International Standards for Neurological Classification of Spinal Cord Injury: Revised 2019,” Topics in Spinal Cord Injury Rehabilitation
27, no. 2 (2021): 1–22, 10.46292/sci2702-1.PMC815217134108832

### 5.10 Management of Burns and Wounds

Burns patients are especially prone to compromise of the airway and respiratory tract, and to fluid loss and hypovolemic shock. These issues are covered in detail in Sections 5.1, 5.2, 5.3 and are not reiterated in Section 5.10, which focuses on care of the burn wound itself. One burn‐specific airway/respiratory tract issue to note is inhalation injury. Bronchoscopy is both diagnostic and therapeutic (including use of inhalation therapies, such as inhaled heparin). These capabilities are considered extended at second and tertiary hospitals.

Cooling of the burn wound to stop the burning process and mitigate wound progression is core at all levels of the health system. This includes knowledge of when and how to cool burn wounds with clean water and when the risk of hypothermia outweighs the benefits of cooling the wound. The capability to assess the depth and extent of a burn wound is deemed core at all levels of the health care system. These issues bear upon subsequent fluid requirements. The only resource needed for such assessment is training and Lund‐Browder type diagrams to aid in estimating extent of burn injuries across age groups.

The capability for wound cleansing and sterile dressing of a burn wound, at least as an adjunct to transfer, is considered core at all levels of the health care system. This implies training and basic sterile dressing materials.

Many burn wounds can be treated definitively with topical antimicrobials and dressings. The capability for this is deemed core at all levels of the health care system. A variety of topical preparations are available for treatment of burn wounds. The *Guidelines for EsTC* do not make an endorsement of any specific preparation.

Debridement of necrotic tissue or external debris of burn wounds is considered core at second‐level and tertiary care hospitals, as is escharotomy (the removal or incision of dead skin in areas of full thickness burns). Capabilities for this imply the training of the clinician and the provision of basic surgical equipment to perform the procedures. These capabilities are deemed extended in some first‐level hospitals, either those with a general surgeon present or in rural, low‐income areas with limited capabilities for referral, if a general practitioner has the skills needed to perform these procedures.

Skin grafting of burn wounds that are not healing in a timely manner is considered core at second‐level and tertiary hospitals. This implies the training of the clinician. It also implies the availability of a dermatome to harvest the graft (including the option of low‐cost equipment for harvesting skin, such as a Humby knife). Similarly, skills for meshing grafts by hand with a scalpel or use of skin mesher are also considered core at these levels. These capabilities are deemed extended in some first‐level hospitals, either those with a general surgeon present or in rural, low‐income areas with limited capabilities for referral, if a general practitioner has the skills needed to perform these procedures.

Full‐thickness burns are associated with the greatest mortality and most disappointing functional results. Early excision of these wounds (with or without immediate grafting) can reduce mortality and improve functional outcome. Such early, aggressive therapy has the possible side‐effect of increasing blood loss and hence mortality if not performed with adequate safeguards for hemostasis (e.g., use of tourniquets, epinephrine clysis solution, electrocautery) and adequate capabilities for fluid and blood resuscitation. For these reasons, it is deemed extended at tertiary and secondary‐hospitals, and not applicable to first‐level hospitals. Use of early excision implies not only surgeons who are trained in the safe performance of the procedure, but also sufficient anesthetic capability to adequately resuscitate patients during and after the procedure. If these cannot be assured, it might nonetheless be possible to perform small scale early excision and grafting on functionally and cosmetically critical areas, such as the face, hands, and over joints. Use of temporary or permanent skin substitutes (e.g., xenograft, allograft, dermal templates) can be used to temporize wounds and the physiology of skin loss, test the wound for autograft readiness, and/or provide a more suitable wound base for autografting to mitigate risks of hypertrophic scar. Skin substitutes are considered extended at tertiary facilities given limited availability and high cost, although there is a growing experience with these in some LMICs [1].

Burn wound contractures of the extremities are a frequent cause of disability in many countries. Most are eminently preventable through improved attention to splinting and positioning plus physiotherapy/occupational therapy during the period of wound healing and scar remodeling (usually 12–18 months after wound closure). Further details of physiotherapy, occupational therapy, and rehabilitation are provided in Section 5.11. However, as regards burns, at least basic expertise in splinting and positioning and in physiotherapy/occupational therapy are deemed core at all hospital levels. The primary resource for this is training. Even if fully trained therapists are not available, the needed expertise could be provided by other hospital staff (nurses, doctors, or other personnel) with supplemental training in physiotherapy/occupational therapy. The only physical resources required are low‐cost splints that could be fashioned from locally available materials, if need be (e.g., plaster of Paris, reusable thermoplastic materials, or other rigid supports).

Reconstructive surgery to correct burn wound contractures of extremities or other body parts, as well as for repair of poor cosmetic results of facial burns, are deemed to be extended at second‐level hospitals and core at tertiary care hospitals.

The general topic of management of wounds is partially considered in Section 5.8 (Management of extremity injury). For the sake of completeness, it is covered more fully here. The capability to assess a wound for its potential for mortality and disability is considered core at all levels of the health care system. Referral to the next highest level of the health care system would ordinarily be expected if a better outcome (both survival and functional status) would be likely to be achieved. Potential for disability includes both damage to underlying nerves, vessels and components of the locomotive system, as well as the extent and location of soft‐tissue defects. Non‐surgical management of uncomplicated wounds consists of cleaning and dressing. The capability for such is deemed core at all levels of the health care system. Minor surgical management of wounds includes minor debridement and suturing. This implies the availability of expertise as well as basic surgical supplies, including anesthetics (primarily local), antiseptics, surgical instruments and suture. These are considered core at all levels of the health care system. Many open wounds in rural low‐income areas are cared for solely at basic/clinic facilities. In such environments, assuring the capability of clinics to care for wounds would be very useful in assuring overall care of injured patients.

Major surgical management of complicated wounds includes extensive debridement and repair of injured structures, as indicated. It often implies repeat procedures and skin grafting. This capability is deemed core at second‐level and tertiary care hospitals. It is deemed as extended at first‐level hospitals. As indicated in the section on open fractures, the initial management of such complicated wounds is often undertaken at first‐level hospitals for a period of hours to days before transport for referral can be arranged. Under these circumstances, assuring adequate early management of complicated wounds, with or without underlying open fractures, would be very useful in assuring adequate care of severely injured patients. The spectrum of training for management of such wounds includes a knowledge of damage control principles and when not to close wounds in cases where they are too severely contaminated.

The capability for tetanus prophylaxis implies the training to categorize a wound by its tetanus risk and to know the required tetanus prophylaxis based on local epidemiology (e.g., status of immunization of the population). This capability also implies the availability of both tetanus toxoid and tetanus antiserum. These are deemed core at all levels of the health care system.

**TABLE 10:** Burns and wounds.

Resources	Facility level			
	Clinic	First	Second	Tertiary
Burns				
Bronchoscopy	NA	NA	Ex	Ex
Assessment of depth and extent	Co	Co	Co	Co
First aid with cool running water	Co	Co	Co	Co
Sterile dressings	Co	Co	Co	Co
Topical antibiotic dressings	Ex	Co	Co	Co
Debridement	NA	Ex	Co	Co
Escharotomy	NA	Ex	Co	Co
Skin graft	NA	Ex	Co	Co
Early excision (with or without immediate grafting)	NA	NA	Ex	Ex
Skin substitutes	NA	NA	NA	Ex
Physiotherapy/occupational therapy and splints to prevent contractures in burn wounds	NA	Co	Co	Co
Reconstructive surgery	NA	NA	Ex	Co
Wounds				
Assess wounds for potential mortality and disability	Co	Co	Co	Co
Non‐surgical management: Clean and dress	Co	Co	Co	Co
Minor surgical: Clean, suture	Co	Co	Co	Co
Major surgical debridement and repair	NA	Ex	Co	Co
Tetanus prophylaxis (toxoid)	Co^a^	Co	Co	Co
Tetanus prophylaxis (immunoglobulin)	NA	Co	Co	Co

^a^ Tetanus toxoid should be core at any clinic facility at which there is refrigeration.

Reference for Section 5.101

J. L.
Roberson
, 
J.
Pham
, 
J.
Shen
, et al., “Lessons Learned From Implementation and Management of Skin Allograft Banking Programs in Low‐ and Middle‐Income Countries: A Systematic Review,” Journal of Burn Care & Research
41, no. 6 (2020): 1271–1278, 10.1093/jbcr/iraa093.32504535


### 5.11 Rehabilitation

As noted in the introductory sections of these *Guidelines,* there is a vast amount of preventable injury‐related disability, especially due to fractures and burns of the extremities. Efforts to prevent such disabilities are needed in acute care, as covered in the preceding Section 5.8 (Management of extremity injury) and Section 5.10 (Management of burns and wounds). Efforts are also needed in the rehabilitation of people with injuries after the acute treatment phase is over, to maximize recovery of independent function. Rehabilitative services have been considered briefly in some of the preceding sections. The current section covers these more comprehensively for all types of injuries.

The following recommendations concentrate on injuries to the extremities, the anatomic pattern of injury‐related disability that is most common and most likely to be improved through low‐cost improvements in rehabilitation services. Hence, basic physiotherapy/occupational therapy for those recovering from extremity injuries (especially fractures and burns) is deemed core at all hospital levels. This includes such activities as the proper use of splints to prevent burn wound contractures, and range‐of‐motion and strengthening exercises for recovery from all types of extremity injuries. In the light of the fact that many injured patients receive follow‐up care at clinics after hospital discharge, such capabilities are extended at this level of facilities. Obviously, it would be ideal to have fully trained physiotherapists and occupational therapists providing such care at all levels. However, given limitations of cost, appropriate elements of training in physiotherapy/occupational therapy might reasonably be provided to key personnel. This might involve a specifically designated nurse (or other appropriate person) who would take on the role of providing physiotherapy at a first‐level hospital.

The fields of physiotherapy and occupational therapy overlap somewhat and vary between countries. For the purposes of these *Guidelines,* physiotherapy refers to those services needed to improve range of motion, strength and mobility. Occupational therapy refers to those services needed to improve range of motion and strength, specifically for the upper extremities, and to assist patients in regaining independent function for activities of daily living (e.g., dressing, feeding). The latter includes the provision of adaptive devices and training in their use.

The full spectrum of physiotherapy, including that appropriate for patients with injuries of the head and spinal cord, is deemed core at tertiary hospitals and extended at second‐level hospitals. The full spectrum of occupational therapy is deemed extended at second‐level and tertiary hospitals. As indicated above, the provision of fully trained professionals in each field is ideal. However, given limitations of cost, appropriate elements of training in these fields might reasonably be provided to key personnel at each facility as a way of maximizing the availability of such rehabilitation services. The key elements of such care that might be promoted in the face of a lack of fully trained personnel still remain to be defined.

Prosthetic services are deemed core at tertiary and extended at second‐level hospital. These services include the provision of the prostheses themselves, as well as personnel with suitable expertise to fit patients with the prostheses properly and to handle problems that may arise in their use. Given the mental distress of severe injury and the resulting high incidence of post‐injury psychological problems, psychological counseling in some form is deemed core at all hospital levels. This includes capabilities for both screening of injured persons for incipient psychological problems and appropriate treatment. It also includes assisting patients in psychological adjustment to their disabilities. The provision of fully trained mental health workers and psychologists would be ideal. However, given the shortages of such trained personnel, appropriate elements of training in psychological counseling might reasonably be provided to a number of key personnel, such as nursing and medical staff (or other persons with suitable qualifications).

Two additional specialized rehabilitative services include neuropsychology for the diagnosis and treatment of cognitive dysfunction, and speech pathology for the diagnosis and treatment of disorders of communication and swallowing. Both are especially useful in the recovery of patients with traumatic brain injury. These are both deemed extended at second‐level and tertiary hospitals. Fully trained professionals for each field would be ideal. However, given the shortages of such personnel, appropriate elements of training in speech therapy and therapy for cognitive dysfunction might reasonably be provided to a number of key personnel, such as medical and nursing staff (or other suitable persons).

The functional recovery of severely injured or ill patients often involves complicated rehabilitation issues, coordination of the input of multiple professionals, and treatment of ongoing medical problems. The coordination of a multidisciplinary team for rehabilitation is fundamental to adequate rehabilitative recovery of trauma patients. The field of physical medicine and rehabilitation has arisen in response to this need. The specific terminology for this field varies between countries. Regardless of the terminology, fully trained physician‐level specialists in this field are considered core at tertiary hospitals and extended at second‐level hospitals. The low level of availability of physical medicine and rehabilitation specialists worldwide prevents this recommendation from being deemed core more widely. Similar considerations apply to specialized rehabilitation nurses. These personnel have specialized training in the management of severely disabled persons, including neurogenic bladder management, bowel programs, prevention of pressure injury (e.g., ulcers) and monitoring for nosocomial infections. Such expertise is deemed core at tertiary hospitals and extended at second‐level hospitals. Low availability of such expertise prevents this recommendation from being considered core more widely. Ideally, evaluation of a trauma patient for rehabilitation needs should begin when they are still undergoing acute care. Ideally, this should be done by a physical medicine/rehabilitation specialist. When such are not available, evaluation should be done by people with physiotherapy/occupational therapy skills.

A useful adjunct to the work of physical medicine and rehabilitation specialists is electromyography (EMG), which is of benefit in the evaluation and treatment of peripheral nerve injuries. This is deemed extended at second‐level and tertiary facilities.

Finally, many injured persons will never regain the functional status they enjoyed before they were injured. Enabling them to function optimally in society is one of the goals of rehabilitation. This often implies connecting patients who are being discharged from the hospital with community‐based rehabilitation services. The *Guidelines for EsTC* recognize the importance of such services. As the *Guidelines* focus on facility‐based trauma care, further details of the elements of community‐based rehabilitation will not be addressed here. However, a knowledge of existing community services and the capability to assist disabled patients in accessing and utilizing such services after discharge (e.g., discharge planning) are considered core at all hospital levels [1].

**TABLE 11:** Rehabilitation.

Rehabilitation	Facility level			
	Clinic	First	Second	Tertiary
PT/OT for recovery of extremity injuries	Ex	Co	Co	Co
Full spectrum of physiotherapy	NA	NA	Ex	Co
Full spectrum of occupational therapy	NA	NA	Ex	Ex
Prosthetics	NA	NA	Ex	Co
Psychological counseling	Ex	Co	Co	Co
Neuropsychology for cognitive dysfunction	NA	NA	Ex	Ex
Speech pathology	NA	NA	Ex	Ex
Physical medicine and rehabilitation specialist‐level care (or similar physician level specialist in rehabilitation)	NA	NA	Ex	Co
Electromyography	NA	NA	Ex	Ex
Specialized rehabilitative nursing	NA	NA	Ex	Co
Discharge planning	NA	Co	Co	Co

Abbreviation: PT/OT, physiotherapy/occupational therapy.

Reference for Section 5.111
World Health Organization
, 
World Rehabilitation Alliance
 (2026), https://www.who.int/initiatives/world‐rehabilitation‐alliance.

### 5.12 Pain Control and Medicines

Through the work of the Expert Committee on Selection and Use of Essential Medicines, the WHO has developed a *Model List of Essential Medicines* (*MLEM*). The medicines on the MLEM have been selected to address major public health problems, have documented evidence of efficacy and safety, and are cost‐effective [1, 2, 3]. The *MLEM* contains over 500 medicines that the WHO recommends should be available within a functioning health system at all times in adequate amounts, in appropriate dosage forms, with assured quality and at an affordable price. The *MLEM* is updated every 2 years. As with the *Guidelines for EsTC*, the *MLEM* is intended to be adapted on a national basis. Currently, over 150 countries have national essential medicines lists.

Most medications needed for essential trauma care are already included in the *MLEM*. However, their availability is still far from complete, especially in rural areas of low‐income countries [1, 2, 3]. Hence, the *Guidelines for EsTC* lay out some of the most critical trauma‐related medicines from the *MLEM*, in order to promote greater availability of these medicines. The *Guidelines* add further definition as to levels of the health care system at which the various medicines should be considered core, with respect to the care of injured patients. In the accompanying table, medicines are grouped by the categories in the *MLEM*. Not all of the 27 categories of the MLEM are applicable. Only those which are applicable are listed in the *Guidelines*. Within each category, the *Guidelines* address only those medicines which are trauma‐related. Furthermore, in this section, we consider only medicines that are on the *MLEM*. There are other medicines that are in widespread use for trauma care and that may be beneficial that are not on the MLEM, that are not considered herein.

In some cases, the *MLEM* indicates a therapeutic group, in which various drugs could serve as alternatives; these are indicated as “or equivalent” in the *Guidelines for EsTC*. In the *MLEM*, drugs may be included in several categories. For example, diazepam is listed under both anesthetics and anticonvulsants. For brevity, in Table 12, each medicine is listed only once.

The *MLEM* is divided into a core list and a complementary list. The core list contains medicines that are efficacious, safe and cost‐effective for major health problems. The complementary list contains medicines that are also efficacious and safe, but not necessarily as affordable as those on the core list. In the *Guidelines for EsTC*, almost any drug on the complementary list is listed as extended rather than core.

The *Guidelines for EsTC's* list of medicines is not intended to be an exhaustive list of all medicines that might conceivably be needed by trauma patients. For example, in malaria endemic areas, postoperative fever is often due to recurrence of malaria. Antimalarial agents are covered extensively by the *MLEM*, but not by the *Guidelines for EsTC.* Likewise, some medicines have broader indications that would mandate their availability at different levels of the health care system, but their indications and applicability might be different for trauma patients. The *Guidelines for EsTC* address their applicability for trauma, as would be prescribed by a provider at that level. For example, therapeutic foods might reasonably be available at the clinic level. However, these are designated as NA for trauma patients at this level, as trauma patients with malnutrition arising from their injuries would probably necessitate referral to a hospital.

The *MLEM* explains that the selection of these medicines is only one step, which needs to be followed by the appropriate use of these medicines. This implies that individuals who need them receive the correct medicine, in an adequate dose, and at an affordable cost. This depends on factors such as regulatory decisions, procurement, and training. Similar items need to be addressed to promote improved trauma care. In particular, it is to be emphasized that, whatever pharmaceutical agent is being considered, the health care providers utilizing this agent should have sufficient training and skill to administer or prescribe it effectively and safely.

With respect to regulations and procurement, many of the medicines in *MLEM* Section 2 (Pain, fever and inflammation) are subject to international restrictions to prevent illicit narcotic traffic. In some cases, otherwise reasonably stringent international controls prevent effective analgesics from reaching those who need them, especially in rural, low‐income areas. Trauma patients represent one of the largest groups of people in severe pain, and hence one of the largest groups in need of effective, affordable analgesics. Hence, suitably amending existing regulations or otherwise finding ways to assure the availability of inexpensive, effective, narcotic‐level pain‐relieving medications would be a key element in planning for strengthening trauma care.

Most of the medicines in the accompanying table are self‐explanatory or have been addressed in further detail in the *MLEM*. A few points need to be mentioned. Section 1 addresses anesthetics, preoperative medicines and medical gases. The *Guidelines for EsTC* deems medicines and capabilities for general anesthesia as extended for first‐level hospitals. This is because many first‐level hospitals globally currently perform only simple operations under sedation or local anesthetics. Advocacy to promote greater access to surgical care (including for trauma, obstetrical emergencies, and other emergency surgery) has emphasized the important role that first‐level hospitals can play [4, 5]. It would be very reasonable for any country wishing to increase access to trauma and emergency surgery at first‐level hospitals to designate general anesthesia as core at this level. This would be especially the case in environments where distances between first‐level hospitals and higher‐level facilities are high. Designating general anesthesia as core at first‐level hospitals implies not only the availability of the medicines, but also the skills and equipment for administering them and for monitoring (during operations) and post‐operative care of patients receiving general anesthesia. As spinal anesthesia can occasionally require conversion to general anesthesia, spinal anesthesia is also listed as extended at first‐level hospitals. Both are considered core at higher level hospitals. Nitrous oxide is decreasingly being used globally, in part because of its green house gas effect. Because of its good safety profile and because it is still widely in use, it is listed as extended (rather than NA) at all hospital levels. When used, it should be provided by tank, rather than centrally, to decrease its emission into the atmosphere. Muscle relaxants are often used during general anesthesia. These are covered in a later *MLEM* section (Section 20).

Medicines from Section 2 (Pain control and palliation) are also used for palliation. Details on palliative care are beyond the scope of this publication. However, it should be noted that there are often trauma patients with conditions that might warrant palliative care (depending on their age, pre‐existing medical conditions, and prognosis of their injuries), such as extensive burns and severe head injuries, among others. Ethical decisions regarding palliation versus continued definitive and supportive care with low probability of successful outcome of course need to be made within the ethical perspectives of local systems of care and local resource availability. When needed, medicines for palliation should be available [6, 7, 8].

Medications from Section 3 (Anaphylaxis) and Section 4 (Poisonings) of the *MLEM* are included here because of the possible need to treat complications of medicines elsewhere on the list, including allergic reactions to antibiotics and respiratory depression from narcotics.

Under Section 6 (Infections), only those antibiotics which generally pertain to care of the injured are included. Anti‐tuberculosis medications and antimalarial agents (see above) are not included here, although they are in the *MLEM*. The *MLEM* categorizes antibiotics into: (1) access group (access to which should be widely assured); (2) watch group (less wide access primarily to diminish emergence of resistance); and (3) reserve group (restricted access, to be used primarily for multi‐drug resistant organisms, all on complimentary list). In the *Guidelines for EsTC*, all antibiotics on the watch group are considered extended at first‐level hospitals and core at higher level hospitals (other than those antibiotics on the complimentary list, which are only extended).

Antibiotic use should be guided by local epidemiology and resistance patterns [9, 10]. Reserve group antibiotics are primary for treating complications in immunocompromised trauma patients, such as those with ventilator associated pneumonia and intra‐abdominal infections. In general, it would be anticipated that most patients with these complications would be at (or referred to) tertiary or at least second‐level hospitals.

Antifungal agents are deemed core at second‐level and tertiary. They are deemed as extended at first‐level, as, in general, trauma patients who develop fungal infections are usually immunocompromised and should probably be cared for at higher levels.

Medicines affecting the blood (Section 10 of the *MLEM*) include several anti‐coagulants. In general these are listed as NA at clinic, extended at first level (due to need for monitoring, such as by serial prothrombin time and partial thromboplastin time), and core at higher levels. One exception is enoxaparin, which is used for deep venous thrombosis prophylaxis and which usually does not need monitoring, which is deemed core at all hospital levels. Tranexamic acid is listed as extended at clinic level and core at all hospital levels. Many clinics also do deliveries and many of these have tranexamic acid available, which should then be considered as core for trauma patients also.

Blood products and plasma expanders (Section 11 of the *MLEM*), Cardiovascular disorders (Section 12), and Fluid and electrolyte balance (Section 26) are further explained in Section 5.3 of these *Guidelines*, on the topic of circulation. The isotonic crystalloid solutions are critical for resuscitation of hypotensive trauma patients, and their availability and appropriate use should be assured in every hospital where severely injured patients are treated. The *MLEM* lists several options for IV fluids (crystalloids). Use of specific formulations varies in different areas of the world. For simplicity, the *Guidelines* combine the IV fluids into two main categories—those containing glucose and those containing balanced salt solutions—both of which are core at all hospital levels. Potassium replacement for hypokalemia and sodium bicarbonate for treatment of acidosis are both core at all hospital levels.

The *MLEM*'s Section 12 (Medicines for cardiovascular disorders) includes epinephrine. Use of this vasoactive agent is usually performed by continuous IV drip. This is deemed core at tertiary hospitals. It is deemed extended at first‐ and second‐level hospitals because of the cost of the infusion equipment and the need to adequately train both medical and nursing staff in the safe administration of these medications. Epinephrine, however, is considered as core at all hospital levels as a means to treat anaphylaxis and other allergic reactions by single dose administration. Several other vasoactive agents are in common use, but not on the *MLEM*. Given the high number of trauma patients globally who need intensive care and hence need such vasoactive agents, future review and potential inclusion of these agents on the *MLEM* should be pursued.

Diuretics (Section 16 of the *MLEM*) include mannitol. Mannitol is one of the tools used for management of raised intracranial pressure (ICP). Overall and ongoing management of raised ICP mainly takes place in the ICU setting and was considered in Table 4 (head injury), where such management was deemed as extended at second‐level and core at tertiary hospitals. Availability of mannitol for single dose use, such as in the emergency unit setting prior to transfer of patients with evidence of raised ICP, is deemed as core at all hospital levels.

Gastrointestinal medicines (Section 17 of the *MLEM*) are included primarily because of the need for peptic ulcer prophylaxis in severely injured patients. Insulin (Section 18) is included in light of increasing evidence of improved outcome for severely injured patients with tighter blood glucose control.

Calcium gluconate (Section 27 of the *MLEM*) is included for treatment of hypocalcemia. This is especially relevant with massive transfusions.

Finally, an important mode of pain control in patients with extremity injuries consists of splinting and immobilization. These have been addressed in Section 5.8 (Management of extremity injury) and Section 5.9 (Management of spinal injury) of these *Guidelines*.

**TABLE 12:** Medicines.

	Facility level			
	Clinic	First	Second	Tertiary
Anesthetics, preoperative medicines and medical gases (MLEM^a^ Section 1)				
Local anesthetic (lidocaine, bupivacaine or equivalent)	Co	Co	Co	Co
General anesthetic (isoflurane, sevoflurane, ketamine, propofol or equivalent)^b^	NA	Ex	Co	Co
Preoperative medication and sedation (atropine, midazolam, ketamine, propofol or equivalent)	NA	Co	Co	Co
Spinal/regional anesthetic (bupivacaine, lidocaine or equivalent)	NA	Ex	Co	Co
Nitrous oxide	NA	Ex	Ex	Ex
Oxygen	Ex	Co	Co	Co
Medicines for pain and palliative care (MLEM Section 2)				
Morphine (or equivalent)	Ex	Co	Co	Co
Codeine (or equivalent)	Ex	Co	Co	Co
Acetylsalicylic acid	Co	Co	Co	Co
Ibuprofen (or equivalent)	Co	Co	Co	Co
Paracetamol (acetaminophen)	Co	Co	Co	Co
Antiallergics and medicines used in anaphylaxis (MLEM Section 3)				
Dexamethasone, hydrocortisone (or other equivalent steroid)	Co	Co	Co	Co
Epinephrine (adrenaline)	Co	Co	Co	Co
Anti‐histamine: Loratadine (or equivalent)	Co	Co	Co	Co
Antidotes and other substances used in poisonings (MLEM Section 4)				
Naloxone	Ex	Co	Co	Co
Sodium nitrite/sodium thiosulfate/hydroxocobalamin^c^ (cyanide/CO poisoning)	NA	Ex	Co	Co
Medicines for diseases of the nervous system: Antiseizure medicines (MLEM Section 5)				
Diazepam (or equivalent, e.g., lorazepam, midazolam)	Ex	Co	Co	Co
Phenobarbital	Ex	Co	Co	Co
Phenytoin	Ex	Co	Co	Co
Levetiracetam (C)^d^	NA	Ex	Ex	Ex
Anti‐infective medicines (MLEM Section 6)				
6.2.1. Access group antibiotics				
Amoxycillin/ampicillin	Co	Co	Co	Co
Amoxycillin and clavulanic acid	Co	Co	Co	Co
Benzylpenicillin (or equivalent)	Co	Co	Co	Co
Cefalexin	Co	Co	Co	Co
Cefazolin	NA	Co	Co	Co
Chloramphenicol	Ex	Co	Co	Co
Clindamycin	NA	Co	Co	Co
Cloxacillin	Co	Co	Co	Co
Doxycycline	Co	Co	Co	Co
Gentamicin	NA	Co	Co	Co
Metronidazole	Co	Co	Co	Co
Sulfamethoxazole and trimethoprim	Co	Co	Co	Co
Infections (WHO EML Section 6)				
6.2.2. Watch group antibiotics				
Cefixime	Ex	Ex	Co	Co
Ceftazidime (C)^d^	NA	Ex	Ex	Ex
Cefotaxime	NA	Ex	Co	Co
Ceftriaxone	NA	Ex	Co	Co
Cefuroxime	NA	Ex	Co	Co
Ciprofloxacin	Ex	Ex	Co	Co
Meropenem/imipenem and cilastin (C)^d^	NA	Ex	Ex	Ex
Piperacillin + tazobactam	NA	Ex	Co	Co
Vancomycin	NA	Ex	Co	Co
6.2.3. Reserve group antibiotics (all on complementary list)				
Reserve group antibiotics (C)^e^	NA	NA	Ex	Ex
6.3. Antifungal medicines				
Amphotericin	NA	Ex	Co	Co
Fluconazole	NA	Ex	Co	Co
Micafungin (C)^d^	NA	NA	Ex	Ex
Therapeutic foods (MLEM Section 9)				
Ready‐to‐use therapeutic food	NA^f^	Co	Co	Co
Medicines affecting the blood (MLEM Section 10)				
10.1 Antianemia medicines				
Ferrous salt	Co	Co	Co	Co
10.2 Medicines affecting coagulation				
Dabigatran (or alternatives)	NA	Ex	Co	Co
Enoxaparin (or alternatives)	NA	Co	Co	Co
Heparin	NA	Ex	Co	Co
Phytomenadione (vitamin K)	NA	Co	Co	Co
Protamine	NA	Ex	Co	Co
Tranexamic acid	Ex	Co	Co	Co
Warfarin (or alternative)	NA	Ex	Co	Co
Blood products of human origin and plasma substitutes (MLEM Section 11)				
11.1 Blood and blood components				
Cryoprecipitate	NA	Ex	Co	Co
Blood transfusion, including whole blood, packed red blood cells, fresh frozen plasma, and platelets are considered in chapter 3				
11.2.1 Human immunoglobulins				
Anti‐D	NA	Co	Co	Co
Anti‐rabies	NA	Ex	Co	Co
Anti‐tetanus immunoglobulin	NA	Co	Co	Co
11.2.2 Blood coagulation factors				
Coagulation factor IX	NA	Ex	Ex	Ex
Coagulation factor VIII	NA	Ex	Ex	Ex
Cardiovascular medicines (MLEM Section 12)				
Epinephrine (adrenaline)	NA	Co	Co	Co
Dermatological medicines (MLEM Section 13)				
Sulfadiazine	Co	Co	Co	Co
Mupirocin	Co	Co	Co	Co
Diagnostic agents (MLEM Section 14)				
14.1 Ophthalmic medicines				
Fluorescein	Ex	Co	Co	Co
Antiseptics and disinfectants (MLEM Section 15)				
Antiseptics: Chlorhexidine, ethanol, polyvidone or alternatives	Co	Co	Co	Co
Disinfectants: Alcohol based hand rub, chlorine base compound, chloroxylenol, glutaral or alternatives	Co	Co	Co	Co
Diuretics (MLEM Section 16)				
Furosemide (or equivalent)	Ex	Co	Co	Co
Mannitol	Ex	Co	Co	Co
Gastrointestinal medicines (MLEM Section 17)				
17.1 Antiulcer medicines				
A02BC proton pump inhibitors and/or A02BA H2‐receptor antagonists	NA^f^	Co	Co	Co
17.2 Antiemetic medicines				
Dexamethasone	NA	Co	Co	Co
Metoclopramide	Co	Co	Co	Co
Ondansetron	NA	Co	Co	Co
17.5.1 Oral rehydration				
Oral rehydration salts	Co	Co	Co	Co
Medicines for endocrine disorders (MLEM Section 18)				
Insulin	NA	Co	Co	Co
Immunologicals (MLEM Section 19)				
19.2 Sera, immunoglobulins and monoclonal antibodies				
Antivenom immunoglobulin (exact type defined locally) (co if required by local epidemiology)	Ex	Co	Co	Co
19.3 Vaccines				
Anti‐tetanus vaccine	Co	Co	Co	Co
Muscle relaxants (peripherally‐acting) and cholinesterase inhibitors (MLEM Section 20)^g^				
Atracurium, neostigmine, suxamethonium (or alternatives)	NA	Ex	Co	Co
Vecuronium (C)^d^	NA	Ex	Ex	Ex
Ophthalmological preparations (MLEM Section 21)				
21.1 Anti‐infective agents				
At least one of: Gentamicin, ofloxacin, tetracycline, or equivalents eye solutions/ointments.	Co	Co	Co	Co
21.3. Local anesthetics				
Tetracaine eye drops (or alternate)	NA	Co	Co	Co
Peritoneal dialysis solution (MLEM Section 23)				
Intraperitoneal dialysis solution (C)^d^	NA	Ex	Ex	Ex
Medicines acting on the respiratory tract (MLEM Section 25)				
Glycopyrrolate/glycopyrronium^h^	NA	Ex	Co	Co
Solutions correcting water, electrolyte and acid‐base disturbances (MLEM Section 26)				
Glucose containing IV fluids	Ex	Co	Co	Co
Balanced salt solution	Ex	Co	Co	Co
Potassium chloride solution	Ex	Co	Co	Co
Sodium hydrogen carbonate (sodium bicarbonate)	Ex	Co	Co	Co
Vitamins and minerals (MLEM Section 27)				
Calcium gluconate (C)	NA	Ex	Ex	Ex
Multiple micronutrient powder	NA	Co	Co	Co

^a^ EML: WHO's *Model List of Essential Medicines* (WHO, 2025).

^b^ General anesthesia is often given with muscle relaxants, which are in MLEM Section 20.

^c^ Hydroxocobalamin is included in MLEM Section 10, but listed here with other treatments for poisoning.

^d^ C: WHO's *Complementary model list*.

^e^ *Cefiderocol, ceftazidime + avibactam, ceftolozane + tazobactam, colistin, fosfomycin, linezolid (or alternative), meropenem + vaborbactam, plazomicin, polymyxin B*.

^f^ Has other indications at this level, but would NA for trauma.

^g^ Muscle relaxants are often given with general anesthesia, which is in MLEM Section 1.

^h^ Used with neostigmine (in MLEM Section 20).

References for Section 5.121
World Health Organization
, 
Essential Medicines: Key Facts
 (2024), https://www.who.int/news‐room/fact‐sheets/detail/essential‐medicines.2
World Health Organization
, The Selection and Use of Essential Medicines, 2025: WHO Model List of Essential Medicines, 24th list (World Health Organization, 2025). 10.2471/B09474.3
World Health Organization
, 
Model List of Essential Medicines. Searchable Database
, accessed January 21, 2026, https://list.essentialmeds.org/.4

J. G.
Meara
, 
A. J. M.
Leather
, 
L.
Hagander
, et al., “Global Surgery 2030: Evidence and Solutions for Achieving Health, Welfare, and Economic Development,” Lancet (North American Edition)
386, no. 9993 (2015): 569–624, 10.1016/S0140-6736(15)60160-X.259248345

C. N.
Mock
, 
P.
Donkor
, 
A.
Gawande
, et al., “Essential Surgery: Key Messages of This Volume,” in Disease Control Priorities (Third Edition): Volume 1, Essential Surgery, ed. 

H.
Debas

, 

P.
Donkor

, 

A.
Gawande

, 

D. T.
Jamison

, 

M.
Kruk

, and 

C. N.
Mock

 (World Bank, 2015).267409916

T. C.
Hardcastle
, 
C.
Gaarder
, 
Z.
Balogh
, et al., “Guidelines for Enhanced Recovery After Trauma and Intensive Care (ERATIC): Enhanced Recovery After Surgery (ERAS) Society and International Association of Trauma Surgery and Intensive Care (IATSIC) Recommendations: Paper 1: Initial Care‐Pre and Intraoperative Care Until ICU, Including Non‐Operative Management,” World Journal of Surgery
49, no. 8 (2025): 1997–2028, 10.1002/wjs.70002.40696570
PMC123384467

T. C.
Hardcastle
, 
C.
Gaarder
, 
Z.
Balogh
, et al., “Guidelines for Enhanced Recovery After Trauma and Intensive Care (ERATIC): Enhanced Recovery After Surgery (ERAS) and International Association for Trauma Surgery and Intensive Care (IATSIC) Society Recommendations: Paper 2: Postoperative and Intensive Care Recommendations,” World Journal of Surgery
49, no. 8 (2025): 2029–2054, 10.1002/wjs.70004.40696568
PMC123384498

T. C.
Hardcastle
, 
C.
Gaarder
, 
Z.
Balogh
, et al., “Guidelines for Enhanced Recovery After Trauma and Intensive Care (ERATIC): Enhanced Recovery After Surgery (ERAS) and International Association for Trauma Surgery and Intensive Care (IATSIC) Society Recommendations: Part 3: Trauma Ethics and Systems Aspects,” World Journal of Surgery
49, no. 8 (2025): 2055–2065, 10.1002/wjs.70003.40696567
PMC123384069
World Health Organization
, The WHO AWaRe (Access, Watch, Reserve) Antibiotic Book (World Health Organization, 2022).10
World Health Organization
, WHO Policy Guidance on Integrated Antimicrobial Stewardship Activities (World Health Organization, 2021). https://apps.who.int/iris/handle/10665/341432.

### 5.13 Diagnosis and Monitoring

Equipment and associated expertise for the diagnosis and monitoring of injured patients have been addressed in each of the preceding sections. Because of the overlapping requirements for many of these, all are repeated in this section, along with several that have not previously been discussed. The rationale for most of these items has been discussed above and will not be repeated in full here.

The foundation for the diagnosis and monitoring of injured patients is in adequate clinical examination skills. Basic equipment for the diagnosis of life‐threatening injuries and the monitoring of vital signs include stethoscope, blood pressure (BP) cuff, pocket torch and thermometer, all of which are considered core at all levels of the health care system. Fetal stethoscope and urinary catheter with collection bag for the measurement of urinary output are core at all hospital levels. Some clinics do deliveries and prenatal care. Fetal stethoscopes would be considered as core at such clinics. Electronic cardiac monitoring is core at all hospital levels. Pulse oximetry is core at all levels of the health care system. Assessment of central filling pressures (by either central venous pressure monitoring and/or point of care ultrasound) as an aid to management of shock is deemed core at higher level hospitals and extended at first‐level hospitals.

Plain‐film radiography and portable plain X‐rays (mobile radiography units) are core at all hospital levels. Several more expensive imaging capabilities, including CT scans, contrast radiography, and fluoroscopy/image intensification, often involve fully trained radiologists and are considered core at tertiary hospitals, extended at second‐level hospitals, and mostly NA at first‐level hospitals. Several other expensive imaging capabilities, including angiography (which may include capabilities for interventions such as embolization), MRI and nuclear medicine exams, add value to the management of injured patients. They are listed as extended at various levels, as indicated in the table. Their costs prevent them from being designated core at any level at this time.

Ultrasound examination of the abdomen in trauma is usually performed by focused assessment with sonography for trauma (FAST) and is used to detect the presence of haemoperitoneum. This is considered: extended at first‐level hospitals and core at higher level hospitals. For first‐level hospitals with high volumes of trauma, it would be reasonable to designate ultrasound as core. When utilized, it should be recognized that the skills needed to perform US examination for haemoperitoneum are different than those needed to perform basic obstetric evaluation, which is available in many LMIC environments. When designated core for the evaluation of abdominal trauma, the following need to be assured: 24‐hour availability of the equipment (which implies timely repair of any malfunctioning equipment); 24‐hour availability of staff skilled in the performance of the procedure; and ongoing monitoring of the accuracy of the results of the scans. Ultrasound has other uses in trauma, such as use in obtaining vascular access, diagnosing deep venous thrombosis, and diagnosing vascular injuries (such as blunt carotid or vertebral artery injuries). As with X‐rays, the *Guidelines* do not go into detail on all potential examinations and uses of ultrasound.

The measurement of hemoglobin concentration or hematocrit by any suitable, reliable technique is deemed core at all levels of the health care system, as is the measurement of serum glucose concentration. Gram stains are core at all hospital levels. Bacterial cultures are core at second‐level and tertiary facilities. Measurement of serum electrolytes and serum lactate are core at all hospital levels. Measurement of arterial blood gases are core at second‐level and tertiary hospitals. Measurement of partial thromboplastin time (PTT) and prothrombin time/international normalized ratio are deemed extended at first‐level hospitals and core at higher level hospitals. These are used to monitor the effects of anticoagulant medications (e.g., heparin, warfarin), which were likewise listed as extended at first‐level hospitals and core at higher level hospitals (Table 12). Viscoelastic tests/thromboelastogram can be useful in the management of coagulopathy, such as arises during massive transfusion. These are deemed extended at higher level hospitals. It should be noted that these have not been shown to improve outcomes, but may be useful in decreasing the need for blood products [1, 2, 3, 4, 5].

The use of a pediatric length‐based clinical management support tool (e.g., Broselow or PAWPER tape) is of benefit in the calculation of doses of fluids and medications for children. This is considered extended at first‐level hospitals and core at higher level hospitals. Opthalmoscopes and otoscopes are useful adjuncts for the physical diagnosis of injured patients and are deemed core or extended as indicated in the table. Equipment for the measurement of compartment pressures can be purchased as a ready‐made set or can be constructed using tubing and the gauge from a blood pressure cuff. Expertise to use either one is deemed core at tertiary care facilities.

Any capabilities for monitoring, radiology or laboratory services that are deemed core or that are converted from extended to core in a national plan should meet certain criteria, in addition to mere physical availability of the relevant equipment. These include prompt availability (24 hours a day, 7 days a week, if indicated), sufficient personnel skilled in performing the procedures or tests safely and accurately and in interpreting the results, and, where relevant, sufficient quality assurance mechanisms to monitor the application of the test or procedures.

Equipment should be maintained so as to assure the availability of the related services without interruption due to malfunction. This point is to be emphasized, as the ability to manage and maintain medical equipment has often lagged behind acquisition of the equipment.

**TABLE 13:** Diagnosis and monitoring.

Resources	Facility level			
	Clinic	First	Second	Tertiary
Monitoring				
Stethoscope	Co	Co	Co	Co
Blood pressure cuff	Co	Co	Co	Co
Torch (flashlight)	Co	Co	Co	Co
Thermometer	Co	Co	Co	Co
Fetal stethoscope	Ex	Co	Co	Co
Urinary catheter with collection bag	Ex	Co	Co	Co
Electronic cardiac monitoring	NA	Co	Co	Co
Pulse oximetry	Co	Co	Co	Co
Capnography	NA	Ex	Co	Co
More advanced monitoring (central venous pressure and/or point of care ultrasound)	NA	Ex	Co	Co
Radiological investigations				
Plain films	Ex	Co	Co	Co
Portable plain films	NA	Co	Co	Co
Contrast radiography	NA	NA	Ex	Co
Ultrasound for trauma (including haemoperitoneum)	NA	Ex	Co	Co
CT	NA	Ex	Ex	Co
Angiography	NA	NA	Ex	Ex
Image intensification/fluoroscopy	NA	NA	Ex	Co
MRI	NA	NA	Ex	Ex
Nuclear medicine	NA	NA	Ex	Ex
Laboratory tests				
Haemoglobin/hematocrit	Co	Co	Co	Co
Glucose	Co	Co	Co	Co
Gram stain	NA	Co	Co	Co
Bacterial cultures	NA	Ex	Co	Co
Electrolytes (Na, K, Cl, CO_2_, BUN, creatinine)	NA	Co	Co	Co
Arterial blood gas measurements	NA	Ex	Co	Co
Serum lactate	NA	Co	Co	Co
Partial thromboplastin time (PTT)	NA	Ex	Co	Co
Prothrombin time and international normalized ratio (PT/INR)	NA	Ex	Co	Co
Viscoelastic tests/thromboelastogram	NA	NA	Ex	Ex
Other				
Pediatric length‐based clinical management support tool (e.g., Broselow tape)	Ex	Ex	Co	Co
Otoscope	Ex	Co	Co	Co
Opthalmoscope	Ex	Ex	Co	Co
Compartment pressure measurement	NA	Ex	Ex	Co

Abbreviations: BUN, blood urea nitrogen; CT, computerized axial tomography; MRI, magnetic resonance imaging.

References for Section 5.131

K.
Baksaas‐Aasen
, 
L. S.
Gall
, 
J.
Stensballe
, et al., “Viscoelastic Haemostatic Assay Augmented Protocols for Major Trauma Haemorrhage (ITACTIC): A Randomized, Controlled Trial,” Intensive Care Medicine
47 (2021): 49–59, 10.1007/s00134-020-06266-1.33048195
PMC75508432

C.
Cochrane
, 
S.
Chinna
, 
J. Y.
Um
, et al., “Site‐of‐Care Viscoelastic Assay in Major Trauma Improves Outcomes and Is Cost Neutral Compared With Standard Coagulation Tests,” Diagnostics
10, no. 7 (2020): 486, 10.3390/diagnostics10070486.32708960
PMC74000903

N. P.
Juffermans
 and 
P.
Bouzat
, “Visco‐Elastic Testing in Traumatic Bleeding,” Intensive Care Medicine
50 (2024): 1152–1153, 10.1007/s00134-024-07437-0.38695918
4

V. V.
Kildal
, 
M.
Dahlberg
, 
C. H.
Ek
, et al., “Prognostic Value of Admission ROTEM in Trauma: Enhancing 30‐Day All‐Cause Mortality Prediction Using Machine Learning,” European Journal of Trauma and Emergency Surgery
51, no. 1 (2025): 301, 10.1007/s00068-025-02959-8.41117907
PMC125406305

Z.
Zhu
, 
Y.
Yu
, 
K.
Hong
, 
M.
Luo
, and 
Y.
Ke
, “Utility of Viscoelastic Hemostatic Assay to Guide Hemostatic Resuscitation in Trauma Patients: A Systematic Review,” World Journal of Emergency Surgery
17, no. 1 (2022): 48, 10.1186/s13017-022-00454-8.36100918
PMC9472418

### 5.14 Safety for Health Care Personnel

Given the high rates of HIV and other bloodborne pathogens worldwide, it is imperative that health workers be provided with adequate protection. This is generally true, but especially so in trauma care, where blood and other body secretions are abundant and where the hectic nature of resuscitation may lead to an increased propensity to contact. Capabilities for universal precautions are deemed core at all levels of the health care system. This includes the training necessary to apply these. It also implies provision of the necessary materials, including gloves and goggles, gowns for procedures, as well as capabilities for the safe disposal of sharps and biological materials. All of these are considered core at all levels of the health care system. In the course of trauma resuscitation, large volumes of fluids may sometimes come into contact with health workers' skin. In such cases, adequate water‐resistant clothing (e.g., aprons, fluid resistant shoe covers) should be available. These are core at all hospital levels. They are listed as extended at the clinic level, as some such facilities may not care for severely injured patients under such scenarios. Finally, capabilities for anti‐HIV, post‐exposure prophylaxis with anti‐retroviral medications is deemed core at all levels. This would be facilitated by such a reporting system for exposure incidents.

The above issues of safety for health care workers for trauma care are part of the bigger context of overall health and safety issues for health care workers, which are addressed by several WHO publications [1, 2].

Throughout these *Guideline*s, numerous invasive procedures have been discussed. Implicit in all of these is that they be conducted under appropriate sterile conditions. The capability to achieve sterility is likewise implicit whenever these procedures are mentioned. Sterility for procedures is part of a bigger picture of infection control policies, which are covered by WHO publications [3].

**TABLE 14:** Safety for health care.

Safety for health care personnel	Facility level			
	Clinic	First	Second	Tertiary
Training in universal precautions	Co	Co	Co	Co
Gloves	Co	Co	Co	Co
Surgical mask	Co	Co	Co	Co
N95 mask	Co	Co	Co	Co
Goggles/face shields	Co	Co	Co	Co
Sharps disposal	Co	Co	Co	Co
Biological waste disposal	Co	Co	Co	Co
Gowns for procedures	Co	Co	Co	Co
Aprons (impermeable) and fluid resistant shoe covers	Ex	Co	Co	Co
Post‐exposure prophylaxis for HIV	Co	Co	Co	Co

References for Section 5.141
World Health Organization
, Protection of Health and Safety of Health Workers: Checklist for Health Care Facilities (World Health Organization, 2020), Licence: CC BY‐NC‐SA 3.0 IGO, https://iris.who.int/bitstream/handle/10665/334371/9789240011557‐eng.pdf?sequence=1.2
Caring for Those Who Care: Guide for the Development and Implementation of Occupational Health and Safety Programmes for Health Workers (World Health Organization and the International Labour Organization, 2022), Licence: CC BY‐NC‐SA 3.0 IGO, https://iris.who.int/bitstream/handle/10665/351436/9789240040779‐eng.pdf?sequence=1.3
World Health Organization
, 
Infection Prevention and Control
 (2025), https://www.who.int/health‐topics/infection‐prevention‐and‐control#tab=tab_1.

### 5.15 Organizational and Administrative Functions

#### 5.15.1 Quality Improvement

Quality improvement (QI) is a method of improving medical care by monitoring the elements of diagnosis, treatment and outcome. It has also been known as performance improvement and medical audit. It evaluates the performance of both individual providers and the system in which they work. A variety of techniques have been used for QI in trauma care:

- *Morbidity and mortality conferences*: These involve a discussion of deaths and complications in search of preventable factors. Such conferences are utilized in surgery departments around the world, and the peer review process involved in these is the foundation for improvements in medical care through more formal programs for quality improvement. Typically, all types of cases are discussed at these. Busier trauma centers may have specific morbidity and mortality conferences on trauma alone.
- *Preventable death panel reviews*: These employ reviews of deaths, either at an individual hospital or within a given system, looking for deaths which are considered, by consensus, preventable. This may include deaths due to airway obstruction or isolated splenic injuries. These are similar to morbidity and mortality conferences, but with more methodologic rigor.
- *Audit filters*: A number of quality‐of‐care criteria are established. Particular cases that do not meet these criteria are then reviewed on a systematic basis to see whether there was a problem with the quality of medical care delivered. These include such factors as patients with abdominal injuries and hypotension who do not undergo laparotomy within 1 hour of arrival at the emergency unit; patients with epidural or subdural hematoma who do not undergo craniotomy within 4 hours of arrival at an emergency unit; and open fractures which are not debrided within 8 hours of arrival. Among the audit filters are evaluations of unexpected trauma deaths, such as those occurring with low injury severity scores.
- *Complications*: A long list of potential complications may also be tracked as indicators of the quality of care, in similar fashion to the use of audit filters. This process looks for a rate of complications that is higher than would normally be expected. This includes complications such as pneumonia, wound infections, venous thrombosis and urinary tract infections.
- *Risk‐adjusted mortality*: Through this statistical process, hospitals evaluate the percentage of deaths occurring in patients with low injury severity scores or a low probability of death based on a combination of injury severity scores and trauma scores (TRISS methodology) (or other similar statistical methods). This allows the hospitals to compare themselves against predetermined national norms. Hospitals with higher risk‐adjusted death rates may warrant evaluation of the individual unexpected deaths along with evaluation of their systems of care, to identify elements that might be contributing to such higher risk‐adjusted mortality.

For all of the methods noted above, the primary principle is to identify the problems that are arising due to correctable factors. Corrective action is taken to ameliorate these problems. Finally, the effect of these changes is evaluated to assess whether they have been successful in correcting the problem. The last step is known as “closing the loop.”

An extensive body of literature has confirmed the effectiveness of QI for trauma in improving care, lowering mortality, decreasing complications, and improving efficiency and lowering costs. This body of evidence was summarized at the time of the publication of WHO‐IATSIC *Guidelines for Trauma Quality Improvement Programmes* [1, 2]. At the time, most of the evidence was from high‐income countries. Over the past decade, there has been growing body of evidence for the feasibility and effectiveness of trauma QI programs in LMICs. As just two recent examples: use of preventable death panel reviews in Ghana resulted in a decrease in preventable deaths, and especially lowered one of the main previously‐common causes of preventable deaths, those from inadequate resuscitation from shock [3]. A QI program at two trauma centers in India followed several audit filters, such as hourly monitoring of GCS for patients with traumatic brain injury while in the emergency unit and whether senior doctors were made aware of patients with respiratory distress within 5 minutes of initial assessment. During the 4‐year intervention phase, in‐hospital mortality decreased from 32% to 24% [4, 5, 6].

In the *Guidelines for EsTC*, some type of QI activity (i.e., any of the above techniques) that includes trauma patients in deemed core at second‐ and third‐level hospitals. Clinic and first‐level hospitals might have cases that they handle included in a QI program of a higher‐level facility. But, a QI program that they run themselves would be an extended function.

#### 5.15.2 Trauma Registry

A trauma registry is built upon, but is distinct from, the more general medical records system. A trauma registry may be defined as “a disease‐specific collection composed of a file of uniform data elements that describe the injury event, demographics, prehospital information, diagnosis, care, outcomes, and costs of treatment for injured patients” [7]. In most cases it is computerized, permitting ease of analysis and tracking of QI data elements. It is this ease of analysis and ability to track specific data (such as audit filters, process‐of‐care measures, or complications), as well as ability to adjust for severity of injury, that distinguish trauma registries from general medical records systems [1]. There is a growing body of literature documenting use of trauma registries in LMICs [8, 9, 10, 11]. They can add value to quality improvement programs. For example, the above example of use of audit filters in a trauma QI program in India depended on a trauma registry [4, 5, 6]. Nonetheless, they can be labor intensive to implement and run. For this reason, they are deemed core only at tertiary hospitals. They are deemed extended at second‐level hospitals. Second level hospitals with high trauma volumes could reasonably be designated as core for a trauma registry, especially one that is simpler than would be at a tertiary facility.

#### 5.15.3 CPD: Continuing Professional Development

CPD has also been termed CME: continuing medical education; CNE: continuing nursing education. This usually implies short (2–3 day) courses that are taken by providers after they have completed their core training (e.g., nursing school, medical school, internship/housemanship, residency, etc.). These courses are a means to make participants aware of recent updates and serve as refreshers, ensuring that all who care for the injured have a reasonable, uniform, minimum foundation of knowledge and skills. Multiple studies have documented consistent improvement in participants' trauma care knowledge and skills after taking these courses [12, 13]. Despite their utility, trauma CPD courses have been sub‐optimally utilized, especially in low‐income and lower‐middle‐income countries, and especially in public hospitals and in rural areas. This shortcoming is notable for courses for doctors, but is even more pronounced for CPD for nurses [14, 15]. Increasing the use of trauma CPD needs to address both supply (number, geographic distribution, costs) and demand (willingness, interest, ability) of providers to take the courses [16]. The *Guidelines for EsTC* endorse that professionals providing regular trauma care should have taken, at least once, an externally‐validated, standardized CPD course in trauma care or general emergency care, that encompasses trauma and is relevant to their professional background. Examples include (but are not restricted to): Advanced Trauma Life Support (ATLS), Basic Emergency Care (BEC), National Trauma Management Course (NTMC), Primary Trauma Care (PTC), Trauma Nursing Core Course (TNCC). This requirement is considered core at 2nd and 3rd level hospitals and extended at clinics and 1st level hospitals. At first level hospitals that receive large volumes of trauma patients, it should be considered core.

#### 5.15.4 Triage System

Triage system informs the disposition of patients within the hospital and the urgency of care provided. Acuity‐based triage can be defined as “the action of sorting and prioritizing patients based on the estimation of the urgency for intervention” [17]. It can assure the best match between available resources and patient needs [18]. It can apply to prehospital or facility‐based settings. In these *Guidelines* we provide recommendations only on facility‐based trauma triage, including both that for day‐to‐day care and for mass casualty incidents. A variety of tools have been used in different locations and settings [19], including the WHO's Interagency Integrated Triage Tool, which addresses all emergencies, including specific provisions for trauma [17, 20]. In these *Guidelines*, use of a triage system is considered core at all levels of the health care system. It should be noted that for the clinic level that receives only occasional seriously‐injured patients, such a tool/system would be very simple. At the 2nd and 3rd hospital levels, how well the triage system is working (both under‐ and over‐triage) can be a topic monitored by the quality improvement program.

#### 5.15.5 Trauma Team

The resuscitation of severely injured patients usually involves many personnel, and too often takes place in an environment of anxiety and confusion. A well‐planned and organized approach to such patients is fundamental to optimal management.

There is thus a need to assure that the personnel and equipment needed for resuscitation are present and utilized in an optimal fashion. Achieving this goal is assisted by appropriate pre‐planning and coordination among personnel caring for the injured patient in the field, in the emergency unit and elsewhere in the hospital. Such pre‐planning and coordination involve equipment and supplies in the emergency area. However, more than anything else, they involve the organization of personnel as addressed by the concept of the trauma team.

The exact composition of the trauma team varies with local rules, conditions and staffing. However, a key element is organization, with pre‐assigned roles for members of the trauma team and protocols to assure rapid assembly and efficient operation of the trauma team.

In well‐resourced, larger hospitals, typical members of the trauma team include: clearly defined team leader, someone designated to manage the airway (may be a surgeon, anesthetist, emergency physician, or other), assistant doctor (as needed), primary nurse, recorder (usually a nurse, may also take on the duty of primary nurse), airway assistant (respiratory therapist, nurse or other suitable person), X‐ray technician [21, 22]. The above large numbers of team members are, of necessity, smaller in smaller hospitals. In small, 1st level hospitals in less resourced areas, the concept of trauma teams is somewhat moot, as often only one nurse or one doctor and one nurse provide much of the trauma care [23].

Organized trauma teams have been well documented to improve the process and outcome of trauma care, especially in studies from high‐income countries [24, 25, 26, 27, 28, 29, 30]. There is a growing experience with trauma teams in LMICs [31, 32, 33], with one study reporting a decrease in mortality for injured patients after implementation of trauma team [34].

In the *Guidelines for EsTC*, locally‐appropriate trauma teams with pre‐assigned roles and duties are considered core for 2nd and 3rd level hospitals. They are extended at first level hospitals, where there are less staff. In larger 1st level hospitals that have a large trauma volume, it would be reasonable to designate trauma teams as core.

#### 5.15.6 Tools: Checklists and Forms

Several tools can improve the delivery of initial trauma care and subsequent operative care. These include the Trauma Care Checklist, the Surgical Safety Checklist, and WHO's standardized clinical forms [35, 36]. All are intended to be adapted as needed to suit local needs. Standardized clinical forms imply forms with designated places for information to be recorded, often with memory prompts, as opposed to blank pieces of paper for recording information. There is a strong evidence base for all of these with documented improvements in process of care and decreases in trauma mortality [37, 38, 39, 40]. Clinical forms with built‐in memory prompts have been shown to be a way to improve care in small rural hospitals where limited numbers of staff preclude formal trauma teams [37]. All of these tools (adapted as needed for local circumstances) are considered core at all hospital levels.

#### 5.15.7 Protocols

In the literature, protocols, algorithms, guidelines, and standard operating procedures refer to several overlapping methods to promote (and sometimes require) adherence to standards for good clinical practice by practitioners at a given institution. In this section, we consider “protocols” with the implication that hospital (or other facility) leaders require and may monitor their use, which is to be distinguished from sharing information and loosely encouraging adherence. Examples include (among many) protocols for spinal immobilization and clearance, pediatric or geriatric trauma, chest tube (drain) placement, burn care, transfers, massive transfusions, and many aspects of intensive care unit care.

There is a considerable evidence base for specific protocols and for use of bundles of protocols, mostly from high‐income countries. Many such protocols have been found to improve process of care and to lower mortality [41, 42, 43, 44]. Consequently, some high‐income countries have required hospitals to develop, use, and monitor adherence to a variety of protocols, as part of trauma center accreditation [45]. The evidence base on protocols in LMICs is very limited. A few studies do show several sets of protocols to be feasible and effective (including mortality reductions) [46, 47]. However, this evidence base is limited and data are lacking on feasibility and effectiveness of instituting protocols widely, including cost, cost‐effectiveness, and opportunity costs. Opportunity costs include the time, energy, and expense that might be required to develop locally‐relevant protocols and to monitor adherence. Hence, the *Guidelines for EsTC* deem protocols on clinical care to be extended at all levels of the health care system. Any protocols for trauma care at the clinic level would likely be addressed toward emergency care more generally and would need to be fairly simple.

**TABLE 15:** Organizational and administrative functions.

	Facility level			
	Clinic	First	Second	Tertiary
Policies				
Quality improvement program that includes trauma patients	Ex	Ex	Co	Co
Trauma registry	NA	NA	Ex	Co
CPD in trauma required for providers	Ex	Ex	Co	Co
Triage system in use	Ex	Co	Co	Co
Trauma team with pre‐assigned roles	NA	Ex	Co	Co
Tools				
WHO Trauma Care Checklist in use	Ex	Co	Co	Co
WHO Surgical Safety Checklist in use	NA	Co	Co	Co
Standardized clinical form used for initial management of trauma patients	Ex	Co	Co	Co
Protocols				
Protocols for clinical care	Ex	Ex	Ex	Ex

Abbreviation: CPD, continuing professional development.

References for Section 5.151
World Health Organization
, Guidelines for Trauma Quality Improvement Programmes (WHO, 2009).2

C. J.
Juillard
, 
C.
Mock
, 
J.
Goosen
, et al., “Establishing the Evidence Base for Trauma Quality Improvement: A Collaborative WHO‐IATSIC Review,” World Journal of Surgery
33, no. 5 (2009): 1075–1086, 10.1007/s00268-009-9959-8.19290573
3

D.
Konadu‐Yeboah
, 
K.
Kwasi
, 
P.
Donkor
, et al., “Preventable Trauma Deaths and Corrective Actions to Prevent Them: A 10‐Year Comparative Study at the Komfo Anokye Teaching Hospital, Kumasi, Ghana,” World Journal of Surgery
44, no. 11 (2020): 3643–3650, 10.1007/s00268-020-05683-z.32661695
PMC75299934

J.
Berg
, 
H. M.
Alvesson
, 
N.
Roy
, et al., “Perceived Usefulness of Trauma Audit Filters in Urban India: A Mixed‐Methods Multicentre Delphi Study Comparing Filters From the WHO and Low and Middle‐Income Countries,” BMJ Open
12, no. 6 (2022): e059948, 10.1136/bmjopen-2021-059948.PMC9185581356802715

J.
Berg
, 
S.
David
, 
G.
Bakhshi
, et al., “Effects of Trauma Quality Improvement Program Implementation on Mortality: A Multi‐Center Controlled Interrupted Time‐Series Study,” medRxiv (2024).6

E.
Kapitan
, 
J.
Berg
, 
S.
David
, et al., “The Effect of Trauma Quality Improvement Programme Implementation on Quality of Life Among Trauma Patients in Urban India,” Injury
56, no. 6 (2025): 112333, 10.1016/j.injury.2025.112333.40311153
7
American College of Surgeons
, Resources for Optimal Care of the Injured Patient (American College of Surgeons, 2006).101341148

M. P.
Cote
, 
R.
Hamzah
, 
I. G.
Alty
, et al., “Current Status of Implementation of Trauma Registries' in LMICs & Facilitators to Implementation Barriers: A Literature Review & Consultation,” Indian Journal of Medical Research
159, no. 3/4 (March/April 2024): 322–330, 10.25259/IJMR_2420_23.39361796
PMC114138819

T.
Miclau
, 
Z. J.
Balogh
, 
K. R.
Miclau
, et al., “Trauma Systems: A Global Comparison,” supplement, OTA International
8, no. S3 (2025): e376, 10.1097/OI9.0000000000000376.40321463
PMC1204529510

G. M.
O'Reilly
, 
P. A.
Cameron
, and 
M.
Joshipura
, “Global Trauma Registry Mapping: A Scoping Review,” Injury
43, no. 7 (2012): 1148–1153, 10.1016/j.injury.2012.03.003.22483995
11

G. M.
O'Reilly
, 
B.
Gabbe
, and 
P. A.
Cameron
, “Trauma Registry Methodology: A Survey of Trauma Registry Custodians to Determine Current Approaches,” Injury
46, no. 2 (2015): 201–206, 10.1016/j.injury.2014.09.010.25282299
12

M.
Kadhum
, 
P.
Sinclair
, and 
C.
Lavy
, “Are Primary Trauma Care (PTC) Courses Beneficial in Low‐ and Middle‐Income Countries – A Systematic Review,” Injury
51, no. 2 (2020): 136–141, 10.1016/j.injury.2019.10.084.31679834
13

S.
Debrah
, 
P.
Donkor
, 
C.
Mock
, et al., “Increasing the Use of Continuing Professional Development Courses to Strengthen Trauma Care in Ghana,” Ghana Medical Journal
54, no. 3 (2020): 197–200, 10.4314/gmj.v54i3.11.33883765
PMC804279414

A.
Mohammad
, 
F.
Branicki
, and 
F. M.
Abu‐Zidan
, “Educational and Clinical Impact of Advanced Trauma Life Support (ATLS) Courses: A Systematic Review,” World Journal of Surgery
38, no. 2 (2014): 322–329, 10.1007/s00268-013-2294-0.24136720
15

C.
Mock
, 
S.
Nguyen
, 
R.
Quansah
, et al., “Evaluation of Trauma Care Capabilities in Four Countries Using the WHO‐IATSIC Guidelines for Essential Trauma Care,” World Journal of Surgery
30, no. 6 (2006): 946–956, 10.1007/s00268-005-0768-4.16736320
16
International Association for Trauma Surgery and Intensive Care (IATSIC)
, 
Statement on Trauma CME/CPD Courses
 (2022), https://www.iatsic.org/gallery/IATSIC%20statement%20on%20trauma%20CME%201‐Dec‐2022.pdf2022.pdf.17
World Health Organization
, 
Interagency Integrated Triage Tool
, https://www.who.int/tools/triage.18
World Health Organization
, 
Basic Emergency Care Course
 (2026), https://www.who.int/teams/integrated‐health‐services/clinical‐services‐and‐systems/emergency‐and‐critical‐care/bec.19

Ciesla
D. J.
, 
Kerwin
A. J.
, 
Tepas
J. J.
. Trauma Systems, Triage, and Transport, Chapter 4 in Trauma, ed. 

D. V.
Feliciano

, 

K. L.
Mattox

, 

E. E.
Moore

, 9th ed. (McGraw Hill, 2021), 47–70.20

R.
Mitchell
, 
J. J.
McKup
, 
C.
Banks
, et al., “Validity and Reliability of the Interagency Integrated Triage Tool in a Regional Emergency Department in Papua New Guinea,” Emergency Medicine Australasia
34, no. 1 (2022): 99–107, 10.1111/1742-6723.13877.34628718
21
American College of Surgeons
, Advanced Trauma Life Support. Student Course Manual, 10th ed. (ACS, 2018).22

A. D.
Fox
 and 
D. H.
Livingston
, “Initial Assessment,” Chapter 13 in Trauma, ed. 

D. V.
Feliciano

, 

K. L.
Mattox

, and 

E. E.
Moore

, 9th ed. (McGraw Hill, 2021), 201–213.23

A.
Gyedu
, 
B. T.
Stewart
, 
E.
Nakua
, and 
P.
Donkor
, “Standardized Trauma Intake Form With Clinical Decision Support Prompts Improves Care and Reduces Mortality for Seriously Injured Patients in Non‐Tertiary Hospitals in Ghana: Stepped‐Wedge Cluster Randomized Trial,” British Journal of Surgery
110, no. 11 (2023): 1473–1481, 10.1093/bjs/znad253.37612450
PMC1056440024

B. M.
Dennis
, 
M. A.
Vella
, 
O. L.
Gunter
, et al., “Rural Trauma Team Development Course Decreases Time to Transfer for Trauma Patients,” Journal of Trauma and Acute Care Surgery
81, no. 4 (2016): 632–637, PMID: 27438684, 10.1097/TA.0000000000001188.27438684
25

P. A.
Driscoll
 and 
C. A.
Vincent
, “Organizing an Efficient Trauma Team,” Injury
23 (1992): 107–110, 10.1016/0020-1383(92)90043-r.1572704
26

M.
Noonan
, 
A.
Olaussen
, 
J.
Mathew
, 
B.
Mitra
, 
D. V.
Smit
, and 
M.
Fitzgerald
, “What Is the Clinical Evidence Supporting Trauma Team Training (TTT): A Systematic Review and Meta‐Analysis,” Medicina
55, no. 9 (2019): 551, 10.3390/medicina55090551.31480360
PMC678065127

J.
Pemberton
, 
M.
Rambaran
, and 
B. H.
Cameron
, “Evaluating the Long‐Term Impact of the Trauma Team Training Course in Guyana: An Explanatory Mixed‐Methods Approach,” Americas Journal of Surgery
205, no. 2 (2013): 119–124, PMID: 23246285, 10.1016/j.amjsurg.2012.08.004.2324628528

A.
Sakellariou
, 
P.
McDonald
, and 
R.
Lane
, “The Trauma Team Concept and Its Implementation in a District General Hospital,” Annals of the Royal College of Surgeons of England
77 (1995): 45–52.7717645
PMC250248929

M. A.
Vella
, 
A.
Zone
, 
B.
Succar
, et al., “Teamwork Matters: The Association Between Nontechnical Skills and Cardiac Arrest in Trauma Patients Presenting With Hypotension,” Surgery
175, no. 6 (2024): 1595–1599, 10.1016/j.surg.2024.02.004.38472080
30

D. D.
Vernon
, et al., “Effect of a Pediatric Trauma Response Team on Emergency Department Treatment Time and Mortality of Pediatric Trauma Victims,” Pediatrics
103 (1999): 20–24, 10.1542/peds.103.1.20.9917434
31

S.
Bergman
, 
D.
Deckelbaum
, 
R.
Lett
, et al., “Assessing the Impact of the Trauma Team Training Program in Tanzania,” Journal of Trauma and Acute Care Surgery
65, no. 4 (2008): 879, 10.1097/TA.0b013e318184a9fe.1884980632

R. T.
Petroze
, 
J. C.
Byiringiro
, 
G.
Ntakiyiruta
, et al., “Can Focused Trauma Education Initiatives Reduce Mortality or Improve Resource Utilization in a Low‐Resource Setting?,” World Journal of Surgery
39 (2015): 926–933, 10.1007/s00268-014-2899-y.25479817
PMC470040133

F.
Uribe‐Buritica
, 
S. M.
Carvajal
, 
N.
Torres
, 
L. A.
Bustamante
, and 
A.
García
, “Trauma Teams: Global Reality and Implementation in a Developing Country. Narrative Description,” Revista Colombiana de Cirugía
36, no. 1 (2021): 42–50, 10.30944/20117582.650.34

S.
Carvajal
, 
F. L.
Uribe‐Buritica
, 
A. M.
Ángel‐Isaza
, et al., “Trauma Team Conformation in a War‐Influenced Middle‐Income Country in South America: Is It Possible?,” International Journal of Emergency Medicine
13, no. 1 (2020): 36, 10.1186/s12245-020-00297-7.32664900
PMC736256935
World Health Organization
, 
Clinical Checklists
, accessed, January 21, 2026, https://www.who.int/tools/clinical‐checklists.36
World Health Organization
, 
Emergency Care Toolkit
, (2026), https://www.who.int/teams/integrated‐health‐services/clinical‐services‐and‐systems/emergency‐and‐critical‐care/emergency‐care‐toolkit.37

A.
Gyedu
, 
F.
Amponsah‐Manu
, 
K.
Awuku
, et al., “Differences in Trauma Care Between District and Regional Hospitals and Impact of a Trauma Intake Form With Decision Support Prompts in Ghana: A Stepped‐Wedge Cluster Randomized Trial,” World Journal of Surgery
48, no. 3 (2024): 527–539, 10.1002/wjs.12082.38312029
PMC1096094438

A. B.
Haynes
, 
T. G.
Weiser
, 
W. R.
Berry
, et al., “A Surgical Safety Checklist to Reduce Morbidity and Mortality in a Global Population,” New England Journal of Medicine
360, no. 5 (2009): 491–499, 10.1056/NEJMsa0810119.19144931
39

A.
Lashoher
, 
E. B.
Schneider
, 
C.
Juillard
, et al., “Implementation of the World Health Organization Trauma Care Checklist Program in 11 Centers Across Multiple Economic Strata: Effect on Care Process Measures,” World Journal of Surgery
41, no. 4 (April 2017): 954–962, PMID: 27800590, 10.1007/s00268-016-3759-8.27800590
40

K.
Werner
, 
N.
Risko
, 
J.
Kalanzi
, 
L. A.
Wallis
, and 
T. A.
Reynolds
, “Cost‐Effectiveness Analysis of the Multi‐Strategy WHO Emergency Care Toolkit in Regional Referral Hospitals in Uganda,” PLoS One
17, no. 12 (2022): e0279074, 10.1371/journal.pone.0279074.36516176
PMC975000341

J.
Cuschieri
, 
J. L.
Johnson
, 
J.
Sperry
, et al., “Benchmarking Outcomes in the Critically Injured Trauma Patient and the Effect of Implementing Standard Operating Procedures,” Annals of Surgery
255, no. 5 (2012): 993–999, 10.1097/SLA.0b013e31824f1ebc.22470077
PMC332779142

C. A.
Dai
, 
C. J.
Fang
, 
D.
Schwartz
, et al., “Standardized Protocol for Chest Tube Management for Trauma Patients Significantly Decreases Complications,” Surgery Research and Practice
21 (2023): 2615557, 10.1155/2023/2615557.PMC105390963778013643

E.
Meneses
, 
D.
Boneva
, 
M.
McKenney
, and 
A.
Elkbuli
, “Massive Transfusion Protocol in Adult Trauma Population,” The American Journal of Emergency Medicine
38, no. 12 (2020): 2661–2666, 10.1016/j.ajem.2020.07.041.33071074
44

T. E.
Wurmb
, 
P.
Frühwald
, 
J.
Knuepffer
, et al., “Application of Standard Operating Procedures Accelerates the Process of Trauma Care in Patients With Multiple Injuries,” European Journal of Emergency Medicine
15, no. 6 (2008): 311–317, 10.1097/MEJ.0b013e3283036ce6.19078832
45
American College of Surgeons
, Resources for Optimal Care of the Injured Patient (2022 Standards. Revised 2025, American College of Surgeons, 2025).46

M. R.
Kesinger
, 
J. C.
Puyana
, and 
A. M.
Rubiano
, “Improving Trauma Care in Low‐ and Middle‐Income Countries by Implementing a Standardized Trauma Protocol,” World Journal of Surgery
38, no. 8 (2014): 1869–1874, 10.1007/s00268-014-2534-y.24682314
47

P.
Wuthisuthimethawee
, 
W.
Sookmee
, and 
S.
Damnoi
, “Non‐Randomized Comparative Study on the Efficacy of a Trauma Protocol in the Emergency Department,” Chinese Journal of Traumatology
22, no. 4 (2019): 207–211, 10.1016/j.cjtee.2019.04.003.31208792
PMC6667989

### 5.16 Special Considerations for Children

Almost all of the preceding sections require some amendment for children. These amendments concern particular skills in handling injured children, equipment in pediatric sizes, and adjustment of doses of medications. A brief summary of these issues follows, emphasizing items that might need to be amended to further optimize care of the injured child. These apply to items that have already been designated as either core or extended.

*Airway management (see also Section* *5.1* *)*

Skills:

- recognition of the differences in airway anatomy in children
- somewhat different techniques needed, particularly for endotracheal intubation

Equipment:

- pediatric sizes for nasal and oral airways, bag–valve–masks, laryngoscopes and endotracheal tubes

*Management of respiratory distress (see also Section* *5.2* *)*

Equipment:

- pediatric‐size equipment for oxygen face masks and for chest tubes

*Management of shock (see also Section* *5.3* *)*

Skills:

- knowledge of different baseline vital signs by age
- knowledge of varying physiological responses to blood loss and varying manifestations of shock in children of different ages
- knowledge of pediatric doses for fluids, both for baseline requirements and for treatment of shock
- knowledge of pediatric doses for blood transfusion for treatment of hemorrhagic shock
- skills in the insertion of pediatric intravenous cannulas, in peripheral cutdown access and in insertion of intraosseous lines

Equipment in pediatric sizes:

- intravenous cannulas
- blood pressure cuff
- urinary catheters
- nasogastric tubes
- intraosseous needle or equivalent
- weighing scale
- iv fluids in pediatric packaging, for example, between 100 or 250 mL saline bag

Laboratory facilities:

- ability to perform laboratory tests on small samples of blood from pediatric patients

*Head injury (see also Section* *5.4* *)*

Skills:

- ability to calculate modified Glasgow coma scale for young children

*Extremity injury (see also Section* *5.8* *)*

Skills:

- understanding of specific pediatric orthopedic injuries which are highly prone to disability
- management of fractures specific to the pediatric age group (e.g., epiphyseal fractures)

*Spinal injury (see also Section* *5.9* *)*

Skills:

- knowledge of the varying anatomy of the childhood spine
- interpretation of spinal X‐ray films (required for both non‐surgical and surgical management)

Equipment:

- C‐collars and other materials for spinal immobilization in pediatric sizes

*Burns and wounds (see also Section* *5.10* *)*

Skills:

- assessment of percentage body surface area of burn wounds in young children

*Rehabilitation (see also Section* *5.11* *)*

Skills:

- monitoring of growth and development to assure that the normal milestones are met as closely as possible, despite the injury and any related physical impairment.

*Pain control and medicines (see also Section* *5.12* *)*

Skills:

- knowledge of pediatric doses

Equipment:

- appropriate references or charts to calculate pediatric doses

*Diagnosis and monitoring (see also Section* *5.13* *)*

Equipment:

- urinary catheters in pediatric sizes
- laboratory capabilities for pediatric volumes (as noted above)

## Author Contributions

**Charles Mock:** conceptualization, writing – original draft, writing – review and editing. **Timothy C. Hardcastle:** conceptualization, writing – review and editing. **Christine Gaarder:** conceptualization, writing – review and editing. **Amit Gupta:** writing – review and editing. **Adam Gyedu:** writing – review and editing. **Manjul Joshipura:** writing – review and editing. **Elmin Steyn:** writing – review and editing

## Funding

Charles Mock was supported in part by grant APW 2024/1457410‐0 from the World Health Organization.

## Conflicts of Interest

The authors declare no conflicts of interest.

## Data Availability

The data that supports the findings of this study are available in the appendices of this article.

## Appendix 1 Literature Search for Implementation of *Guidelines for Essential Trauma Care* June 2015–April 2025

**Table:**

A. Al Balushi, M. Al‐Azri, F. Al‐Balushi, H. Al‐Hinai, and Z. Al‐Mandhari, “Evaluating Trauma Care Capabilities Using the Essential Trauma Care Guidelines of the World Health Organization: A Cross‐Sectional Study of Primary Health Centres in Muscat, Oman,” *Sultan Qaboos University Medical Journal* 21, no. 4 (2021): e563–e570, https://doi.org/10.18295/squmj.4.2021.057.
V. Amado, M. T. Couto, M. Filipe, J. Möller, L. Wallis, and L. Laflamme, “Assessment of Critical Resource Gaps in Pediatric Injury Care in Mozambique’s Four Largest Hospitals,” *PLoS One* 18, no. 6 (2023): e0286288, https://doi.org/10.1371/journal.pone.0286288.
V. N. S. Amado, *Pediatric Injury Care in Low‐Resource Hospital Settings: Insights From Mozambique, Before, During and After the COVID‐19 Pandemic (Order No. 30962394)* (ProQuest Dissertations & Theses Global, 2023), 2898718492, https://proxy1.library.jhu.edu/login?url=https://www.proquest.com/dissertations‐theses/pediatric‐injury‐care‐low‐resource‐hospital/docview/2898718492/se‐2.
J. Ankomah, B. T. Stewart, S. Oppong, et al., “Strategic Assessment of the Availability of Pediatric Trauma Care Equipment, Technology and Supplies in Ghana,” *Journal of Pediatric Surgery* 50, no. 11 (2015): 1922–1927, https://doi.org/10.1016/j.jpedsurg.2015.03.047.
B. V. Babu, K. Viswanathan, A. Ramesh, et al., “An Interventional Study on Comprehensive Emergency Care and Trauma Registry for Road Traffic Injuries in India: A Protocol,” *Advanced Journal of Emergency Medicine* 3, no. 4 (2019): e50, https://doi.org/10.22114/ajem.v0i0.232.
B. V. Babu, D. M. Satapathy, R. M. Tripathy, et al., “Trauma Care in India: Capacity Assessment Survey From Five Centers,” *Journal of Surgery Science* 124, no. 3 (2020): 685–690, https://doi.org/10.1016/j.jss.2020.03.002.
K. J. Blair, V. Tushabe, and M. G. Shrime, “Surgical and Trauma Care in Low‐ and Middle‐Income Countries: A Review of Capacity Assessments,” *Journal of Surgical Research* 210 (2017): 37–46, https://doi.org/10.1016/j.jss.2016.11.005.
J. Fuhs, A. Peña, M. Reyes, and T. R. Dillingham, “Assessment of Rehabilitation Infrastructure in Peru,” *Archives of Physical Medicine and Rehabilitation* 99, no. 2 (2018): 353–358, https://doi.org/10.1016/j.apmr.2017.10.020.
P. Hogan, G. O’Reilly, M. Naidoo, et al., “Trauma Registry Implementation in a Western Kenya Referral Centre: Highlighting the QI Approach [Conference Abstract],” Presented at African Conference on Emergency Medicine (AfCEM) (2019), https://www.researchgate.net/publication/331532858_Trauma_Registry_Implementation_in_a_Western_Kenya_Referral_Centre_Highlighting_the_QI_Approach.
R. Khalil, U. Khan, J. A. Razzak, et al., “Assessment of Trauma Care Capacity in Karachi, Pakistan: Toward an Integrated Trauma Care System,” *World Journal of Surgery* 45, no. 7 (2021): 2130–2137, https://doi.org/10.1007/s00268‐021‐06234‐w.
R. Latifi, A. El‐Menyar, R. Consunji, et al., “The Readiness of Emergency and Trauma Care in Low‐ and Middle‐Income Countries: A Cross‐Sectional Descriptive Study of 42 Public Hospitals in Albania,” *International Journal of Emergency Medicine* 9, no. 1 (2016): 7, https://doi.org/10.1186/s12245‐016‐0124‐5.
S. Lombardo, S. M. Wren, C. Lewis, et al., “Trauma Care in Mongolia: INTACT Evaluation and Recommendations for Improvement,” *World Journal of Surgery* 42, no. 10 (2018): 3246–3253, https://doi.org/10.1007/s00268‐018‐4462‐8.
S. Madha, J. Ali, M. Matsumoto, et al., “A Global Perspective on Military and Civilian Trauma Systems Integration [Abstract],” supplement, *Journal of the American College of Surgeons* 237, no. 4S1 (2023): S50, https://journals‐lww‐com.proxy1.library.jhu.edu/journalacs/fulltext/2023/11001/global_surgery.15.aspx.
M. McCullough, K. Afsana, S. N. Chowdhury, et al., “A National Trauma Capacity Assessment of Haiti,” *Journal of Surgical Research* 200, no. 2 (2016): 514–522, https://doi.org/10.1016/j.jss.2015.10.018.
L. Moore, H. Champion, P. A. Tardif, et al., “Impact of Trauma System Structure on Injury Outcomes: A Systematic Review and Meta‐Analysis,” *World Journal of Surgery* 42, no. 5 (2018): 1327–1339, https://doi.org/10.1007/s00268‐017‐4292‐0.
H. Mousazadeh, M. Zargar, L. Malekyan, et al., “Developing Performance Indicators for Trauma Care: A Four Stage Qualitative Study,” *Trauma Monthly* 25, no. 6 (2020): e102631, https://doi.org/10.30491/tm.2020.213631.1026.
A. E. Muñiz, B. Barlow, J. Espinoza, et al., “Preparing Global Trauma Nurses for Leadership Roles in Global Trauma Systems,” *Journal of Trauma Nursing* 24, no. 5 (2017): 287–291, https://doi.org/10.1097/JTN.0000000000000310.
T. Potokar, R. Bendell, P. Nightingale, et al., “A Comprehensive, Integrated Approach to Quality Improvement and Capacity Building in Burn Care and Prevention in Low and Middle‐Income Countries: An Overview,” *Burns* 46, no. 6 (2020): 1243–1251, https://doi.org/10.1016/j.burns.2020.05.029.
K. Pringle, D. Vitale, S. Shah, et al., “A Short Trauma Course for Physicians in a Resource‐Limited Setting: Is Low‐Cost Simulation Effective?,” *Injury* 46, no. 9 (2015): 1796–1799, https://doi.org/10.1016/j.injury.2015.05.021.
S. Shah, N. Merchant, H. Ahmad, et al., “Assessment of the Availability of Technology for Trauma Care in Nepal,” *Injury* 46, no. 9 (2015): 1800–1806, https://doi.org/10.1016/j.injury.2015.06.012.
B. T. Stewart, A. Gyedu, F. A. Abantanga, J. Ankomah, P. Donkor, and C. Mock, “Strategic Assessment of Trauma Care Capacity in Ghana,” *World Journal of Surgery* 39, no. 10 (2015): 2428–2440, https://doi.org/10.1007/s00268‐015‐3132‐3.
B. T. Stewart, A. Gyedu, F. A. Abantanga, et al., “Orthopaedic Trauma Care Capacity Assessment and Strategic Planning in Ghana: Mapping a Way Forward,” *Journal of Bone and Joint Surgery* 98, no. 13 (2016): e56, https://doi.org/10.2106/JBJS.15.01299.
B. T. Stewart, R. Quansah, A. Gyedu, et al., “Serial Assessment of Trauma Care Capacity in Ghana in 2004 and 2014,” *JAMA Surgery* 151, no. 2 (2016): 164–171, https://doi.org/10.1001/jamasurg.2015.3648.
S. Taylor, *Impact of a Focused Trauma Course on Retention of Provider Skills, Knowledge and Confidence at a Regional Hospital in the Dominican Republic (Order No. 28544565)* (ProQuest Dissertations & Theses Global, 2021), 2547498464, https://proxy1.library.jhu.edu/login?url=https://www.proquest.com/dissertations‐theses/impact‐focused‐trauma‐course‐on‐retention/docview/2547498464/se‐2.
P. S. Uthkarsh, S. Nagesh, V. Devaramane, et al., “Assessment and Availability of Trauma Care Services in a District Hospital of South India: A Field Observational Study,” *International Journal of Health Policy and Management* 5, no. 5 (2016): 301–307, https://pmc‐ncbi‐nlm‐nih‐gov.proxy1.library.jhu.edu/articles/PMC4897990.

## Appendix 2 Detailed List of Equipment and Services for the Management of Airway and Breathing (Both Core and Extended Items)

**Table:**

Level of management	Service	Equipment
Airway		
Basic	Jaw thrust, chin lift, and other basic maneuvers	Tongue depressor
		Oropharyngeal airway (Guedel) (range #000 to 4). Oropharyngeal airways (OPAs)—Sizes: Usually range from 000 (infant) to 6 or 7 (large adult). Size 5 is for XL adult and 6 for XXL adult). Supply should be based on the population. Note that size 5–7 are less commonly used
		Nasopharyngeal airway (NPA), hospitals should have age and body weight appropriate NPA
		NPA sizes are 3.0 mm ID (neonate), 3.5 mm ID (1–6 months), 4.0 mm ID (6–18 months), 4.5 mm ID (18 months–3 years), 5.0 mm (ID 3–6 years), 5.5 mm ID (6–9 years), 6.0 mm ID (9–12 years), 6.5 mm ID (12–14 years)
		Adult range: 6.0–9.0 mm ID (24–36 Fr)
		6.0–7.0 mm for adult females
		7.0–8.0 mm for adult males
	Suction	Manual (bulb syringe, foot pump or hand‐powered suction device)
		Pneumatic (wall)
		Electric
		Yankauer tips
		Suction catheters
		Suction tubing (range #10–16)
Advanced	Endotracheal intubation	ET tubes with ET tube connector (range #3.0 to 8.5 mm internal diameter)
		Laryngoscope (with sufficient range of sizes of blades)
		Sizes: 0, 1, 2, 3, and 4
		Introducing stylet/bougie
	Other advanced	Laryngeal mask airway (LMA)—Age and body weight appropriate LMA size should be available
		Esophageal obturator airway/esophageal gastric obturator airway
		Esophageal–tracheal airway (combitube) (two sizes)
		Fiber‐optic endoscope
		Video laryngoscope
		Transilluminator Magill forceps
	Surgical airway	Needle cricothyroidotomy
		Surgical cricothyroidotomy kit
Breathing		
		Face shield
		Pocket mask
		Self‐inflating bag–valve–mask (pediatric and adult) with reservoir
		Ventilator
		Oxygen
		Wall (with flow meter)—this assumed that there is piped oxygen from the oxygen plants (via Pressure Swing Adsorption (PSA) or Liquid Oxygen systems (LOX systems)
		Oxygen cylinders: prefilled tanks of compressed oxygen (with regulator and flow meter)
		Oxygen Concentrators: electrically powered devices that extract oxygen from ambient air
		Use: Ideal for bedside use in wards or clinics
		Portable Oxygen Concentrators (POCs): Lightweight, battery‐powered concentrators
		Nasal cannula
		Nebulization mask
		Venturi mask
